# Viable protoplast isolation, organelle visualization and transformation of the globally distributed plant pathogen *Phytophthora cinnamomi*

**DOI:** 10.1007/s00709-024-01953-y

**Published:** 2024-05-04

**Authors:** Aayushree Kharel, James Rookes, Mark Ziemann, David Cahill

**Affiliations:** 1 School of Life and Environmental Sciences, Deakin University, Geelong Waurn Ponds Campus, Waurn Ponds, VIC 3216 Australia; 2 Burnet Institute, Melbourne, Australia

**Keywords:** *Phytophthora cinnamomi*, PEG/CaCl_2_ transformation, Protoplast, Fluorescence microscopy, Flow cytometry

## Abstract

*Phytophthora cinnamomi* is an oomycete plant pathogen with a host range of almost 5000 plant species worldwide and therefore poses a serious threat to biodiversity. Omics technology has provided significant progress in our understanding of oomycete biology, however, transformation studies of *Phytophthora* for gene functionalisation are still in their infancy. Only a limited number of *Phytophthora* species have been successfully transformed and gene edited to elucidate the role of particular genes. There is a need to escalate our efforts to understand molecular processes, gene regulation and infection mechanisms of the pathogen to enable us to develop new disease management strategies. The primary obstacle hindering the advancement of transformation studies in *Phytophthora* is their challenging and unique nature, coupled with our limited comprehension of why they remain such an intractable system to work with. In this study, we have identified some of the key factors associated with the recalcitrant nature of *P. cinnamomi*. We have incorporated fluorescence microscopy and flow cytometry along with the organelle-specific dyes, fluorescein diacetate, Hoechst 33342 and MitoTracker™ Red CMXRos, to assess *P. cinnamomi*-derived protoplast populations. This approach has also provided valuable insights into the broader cell biology of *Phytophthora*. Furthermore, we have optimized the crucial steps that allow transformation of *P. cinnamomi* and have generated transformed isolates that express a cyan fluorescent protein, with a transformation efficiency of 19.5%. We therefore provide a platform for these methodologies to be applied for the transformation of other *Phytophthora* species and pave the way for future gene functionalisation studies.

**Supplementary Information:**

The online version contains supplementary material available at 10.1007/s00709-024-01953-y.

## Introduction

Plant disease outbreaks present substantial threats to global food security and environmental sustainability. Among the most economically relevant and environmentally devastating diseases globally are those caused by *Phytophthora* species (Burgess et al. 2021). The genus *Phytophthora* belongs to the phylum Oomycota, within the Kingdom Chromista, and the approximately 200 species comprise some of the most devastating plant pathogens, several of which are distributed globally (Brasier et al. 2022). Late blight of potato (*P. infestans*), soybean stem and root rot (*P. sojae*) and sudden oak death (*P. ramorum*) have significant economic, environmental, and social impact (Bourke 1991; Cooke et al. 2000; Kasuga et al. 2016). *Phytophthora cinnamomi* is arguably the most cosmopolitan and damaging plant pathogen, within the genus (Cahill et al. 2008).

*Phytophthora cinnamomi* causes root rot, has a world-wide distribution, and has become invasive in regions where it was previously not present (Hardham and Blackman 2018). Prime examples of the impact of *P. cinnamomi* include decline of holm oak (*Quercus ilex*) and white oak (*Quercus alba*) in Europe and the USA (McConnell and Balci 2015; Frisullo et al. 2018), and avocado and chestnut, globally (Coffey et al. 1984; Vannini and Vettraino 2001; Engelbrecht et al. 2022). This pathogen is the most reported invasive species within natural ecosystems of Australia (Burgess et al. 2021), with heavy mortality of native plants in the jarrah (*Eucalyptus marginata*) forests of Western Australia (Burgess et al. 2017) and in the dry sclerophyll forests of Eastern Australia (Wilson et al. 2020; Weste and Marks 1974). Traditional methods, such as the use of fungicides, are presently limited in their ability to manage diseases caused by *P. cinnamomi*.

Within the *Phytophthora* genus, *P. cinnamomi* is infamous for its recalcitrance to transformation (Horta et al. 2008; Dai et al. 2021). As such, despite being a ‘biological bulldozer’ (Kamoun et al. 2015) with an extremely large and diverse host range, limited transformation and gene functionalisation studies have been conducted, and current protocols fall short of optimisation and follow-up reproducibility. In light of these challenges, our study fills a crucial void in the literature by focusing on the optimization of transformation of *P. cinnamomi*. While Dai et al. (2021) optimized the enzyme concentration required for protoplast isolation and demonstrated successful transformation using a polyethylene glycol (PEG)-mediated approach, they acknowledged the need for further validation of protocol reproducibility. Recognizing the multifaceted nature of transformation protocols, we aimed to explore additional variables such as starting material, growth media, and regeneration media. Through this comprehensive approach, our research aims to enhance the reproducibility and reliability of *P. cinnamomi* transformation protocols, thereby advancing our understanding and application of this important genetic manipulation technique.

Ongoing research on *Phytophthora*-host interactions, supported by omics data has identified multiple target genes that are key contributors towards the pathogen’s virulence. The relatively recent advent and success of gene modification techniques in some *Phytophthora* species through RNA interference (RNAi) (Cheng et al. 2022) and in vivo transformation followed by CRISPR/Cas gene editing (Fang and Tyler 2016) has opened up pathways for gene functionalisation studies. Despite the importance of gene editing techniques in oomycetes, as emphasized in a recent review by Vink et al. (2023), progress in the use of these approaches has been relatively slow and limited to a few *Phytophthora* species. As such, Ghimire et al. (2022) highlighted the need for transformation protocols to be optimized for different *Phytophthora* species to advance research involving gene editing methods like CRISPR/Cas. As proposed by Kharel et al. (2021), the optimization and advancement of gene editing tools in oomycetes is seen as a key strategy for management of *Phytophthora*-associated diseases.

Earlier studies have also described the transformation of *P. cinnamomi* and *P. infestans* mycelia through microprojectile bombardment (Bailey et al. 1993; Cvitanich and Judelson 2003). This latter approach has not been widely used and studies in filamentous fungi (Meyer et al. 2003; Wang et al. 2017) suggest that it is unsuitable for precise and specific genetic alterations. Successful transformation of *P. capsici*, *P. palmivora* and *P. infestans* has been achieved through electroporation (Evangelisti et al. 2019b; Dong et al. 2015; Huitema et al. 2011) and *Agrobacterium*-mediated transformation of zoospores (Huitema et al. 2011; Dong et al. 2015; Evangelisti et al. 2019a, b; Wang et al. 2023; Wu et al. 2016). Latijnhouwers and Govers (2003) have reported low gene silencing efficiency through electroporation, when compared against PEG-mediated transformation. While electroporation has been widely used for transformation of filamentous fungi, Li et al. (2017) outlined some of the key factors, including the intensity of the electric field, capacitance, as well as external conditions like temperature and composition of the buffer, that are challenging to optimize and will influence transformation efficiency. Similarly, the success of *Agrobacterium*-mediated transformation is influenced by multiple factors, including bacterial density and incubation time, and steps that need to be optimized depending on the oomycete species being used (Wang et al. 2023).

Here, we describe a comprehensive and detailed protocol for production of viable protoplasts of *P. cinnamomi*, with the aim of providing a fundamental platform to establish a well-characterized transformation system. We have further shown the novel application of organelle-specific fluorescent dyes to visualize *P. cinnamomi* organelles. This approach has been subsequently applied to investigate the distribution of organelles within the protoplasts, contributing to our understanding of the processes that may determine the success of transformation. Flow cytometry (FCM) was then used to quantify and characterise large populations of protoplasts. We have determined that combining microscopy and FCM are effective approaches to gain deeper insights into the cellular characteristics of *P. cinnamomi* protoplasts. Finally, the *P. cinnamomi* protoplasts generated from germinated cysts were subjected to transformation through the incorporation of a cyan fluorescent gene.

## Materials and methods

### *Phytophthora cinnamomi* culture and maintenance

*Phytophthora cinnamomi* Rands, isolate Du109 (Deakin University culture collection, A2 mating type) (Pc-WT) was used for all experimental procedures. The isolate was routinely cultured in a 90 mm diameter Petri plate, on 10% clarified V8 agar (cV8) (Miller 1955) [(10% clarified Campbell’s V8 vegetable juice (Camden, NJ, USA), 0.1% CaCO_3_, and 1.5% agar] and grown at 24 °C in the dark.

### Visualization of *P. cinnamomi* with organelle-specific dyes

The hyphae, sporangia, zoospores and germinated cysts of Pc-WT were stained with fluorescein diacetate (FDA) (Sigma Aldrich) to visualize live cells, Hoechst 33342 (Hoechst) (Thermo Fisher Scientific) for nuclear staining, and MitoTracker™ Red CMXRos (MitoTracker) (Thermo Fisher Scientific) for mitochondrial visualization. An FDA stock solution of 1 mg/mL was prepared in acetone and stored at -20 °C. A stock solution of Hoechst at 10 mg/mL was prepared in water and stored at 4 °C. MitoTracker stock of 1 mM was prepared in dimethyl sulfoxide (DMSO) and stored at -20 °C for up to 2 weeks.

To visualize the asexual structures of *P. cinnamomi,* a final concentration of 2 μg/mL FDA, 10 μg/mL Hoechst and 50 nM MitoTracker, was prepared in water and used. To visualize mature hyphae, a plug of *P. cinnamomi* was excised from a 10% cV8 plate and placed in a 1.5 mL centrifuge tube. Individual stains were then added to the tube to a final volume of 1 mL of distilled water. Sporangia and zoospores were produced according to a previously described protocol (Islam et al. 2017). Briefly, six plugs from the outer edge of a plate containing Pc-WT were transferred onto pre-sterilized miracloth (Merck, Germany) placed on top of a 10% cV8 plate. The plate was incubated in the dark at 24 °C for 5 days. The miracloth was transferred to a flask containing 5% cV8 broth and the flask was placed under 3 × 30 W fluorescent lights on an orbital shaker at 110 rpm at 24 °C. After 16 h, the miracloth was rinsed thrice with mineral salt solution (MSS) (13.04 mM Ca(NO_3_)4H_2_O, 12.37 mM MgSO_4_.7H_2_O, 5.04 mM KNO_3_) and the flask was incubated in MSS under previously mentioned conditions for 20 h. The miracloth was then transferred to a 90 mm diameter Petri plate, and rinsed with pre-chilled sterile water. The miracloth was covered with pre-chilled sterile water and the plate was incubated at 4 °C for 1 h in the dark. For sporangia visualization, the cold-shocked plugs were separated from the miracloth and placed in a 1.5 mL centrifuge tube. Individual stains were added to the tube to a final volume of 1 mL of distilled water. For zoospore visualization, the plate with miracloth was removed from 4 °C after an hour of incubation and left at room temperature. Zoospore release was monitored with a microscope, and to 1 mL of zoospore suspension, individual stains were added. For germinated cyst visualization, the zoospores were left at room temperature in water for up to 2 h and to 1 mL of germinated cysts, the stains were added. After addition of stain, all tubes were incubated for 10 min at room temperature before visualization. Slides were viewed under differential interference contrast (DIC) microscopy (Zeiss Axioscope, Zeiss Göttingen, Germany) equipped with Colibri light source and fluorescent stain specific channels. The excitation/emission wavelengths used for the stains were: FDA at 515 nm/531 nm, Hoechst at 353 nm/465 nm and MitoTracker at 578 nm/604 nm. Images were obtained using a digital camera attached to the microscope. For each channel, the light source intensity and exposure time were kept constant, and Zeiss Zen 3.1 software was used to process the images.

### Protoplast isolation and regeneration

#### Preparation of starting material

For isolation of protoplasts, two different starting materials of *P. cinnamomi* were used – hyphae of two different ages and germinated cysts, that were grown in different media.

The media used were 10% cV8 broth, pea broth (McLeod et al. 2008) and rich pea broth (Fang et al. 2017a) without or with β-sitosterol (20 μg/mL final concentration).

#### Mature hyphae

From a 3-day old culture of *P. cinnamomi* grown on 10% cV8 plate, six plugs of 5 mm diameter were excised from the growing edge of the colony and transferred into a flask containing 50 mL of each media and incubated for 18 h or 40 h, at 24 °C in the dark. The mycelial mats were then harvested, rinsed with sterile deionized water and then, 0.8 M mannitol, before incubating in 0.8 M mannitol for 10 min. Mannitol induces plasmolysis which prepares the hyphae for enzyme digestion and protoplast isolation.

#### Germinated cysts

Zoospores of *P. cinnamomi* were produced using the same method described above. Zoospore release from sporangia was monitored, and the suspension was adjusted to a density of 10^5^–10^6^ spores/mL concentration with sterilised distilled water. Zoospore suspension of 5 mL was transferred to a 50 mL centrifuge tube and 15 mL of each media [10% cV8 broth, pea broth and rich pea broth, without or with β-sitosterol (20 μg/mL final concentration)] was added. The tube was gently vortexed for 60 s to induce encystment, resulting in 100% encystment across all types of media, and subsequently, the cysts were incubated in the dark at 24 °C.

After 18 h, the tubes were centrifuged (Beckman Coulter Allegra 6R, United States) at 1000 g for 5 min and the supernatant was discarded. The pellet was washed with water then rinsed with 0.8 M mannitol, before incubating in 0.8 M mannitol for 10 min. The plasmolysed germinated cysts were then used for protoplast isolation.

#### Protoplast isolation

The protocol for producing *P. cinnamomi* protoplasts was similar to previously described methods (Fang et al. 2017a), with modifications. Plasmolysed starting material (hyphae or germinated cysts) was transferred to an enzyme solution containing 5 mg/mL each of cellulase (C224, Phytotechnology Laboratories) and lysing enzymes (L1412-5G, Sigma Aldrich) (Dai et al. 2021). The mycelia were uniformly dispersed in the enzyme solution and incubated on an orbital shaker at 55 rpm, 24 °C for 45 min. The digested products were filtered through miracloth to remove the mycelial debris. The flow-through was collected and centrifuged at 700 g for 4 min to pellet the protoplasts. The supernatant was discarded, and protoplasts were slowly resuspended in 5 mL W5 solution (5 mM KCl, 125 mM CaCl_2_.2H_2_O, 154 mM NaCl, and 177 mM glucose), and it was further made up to 10 mL with the addition of W5 solution, before being centrifuged at 700 g for 4 min. The supernatant was discarded, and the pellet were resuspended in 5 mL W5 solution. The resuspended protoplasts were placed on ice for 30 min, before determining their protoplast density with a hemocytometer.

#### Protoplast count and statistical analysis

The number of protoplasts was calculated using a hemocytometer and the final values were represented as mean ± standard error of the mean. The results are representative of three independent replicates, with three samples per replicate. Statistical analyses were conducted using 2-way ANOVA and Tukey multiple comparison tests.

### Assessing protoplast integrity and viability using fluorescent dyes

#### Microscopy for organelle visualization

The final concentration of fluorescent dyes used for protoplast visualization was 2 μg/mL FDA, 10 μg/mL Hoechst and 50 nM MitoTracker, as mentioned previously. The excitation/emission wavelengths used for the stains were: FDA at 515 nm/531 nm, Hoechst at 353 nm/465 nm and MitoTracker at 578 nm/604 nm. Typically, the dyes used are toxic to the fragile protoplasts, thus, a brief incubation period of 10 min at room temperature was adopted for all staining procedures, and the images were captured immediately thereafter. All microscopy images were obtained using a Zeiss Axioscope (Zeiss, Göttingen, Germany) equipped with Colibri. The images are representative of three biological replicates.

#### Flow cytometry for assessment of protoplast numbers and their characterisation

The stained protoplasts (as prepared above) were analyzed through the use of a flow cytometer (BD FacsCanto II analyze, Becton Dickinson, United States) and data was processed with the FlowJo software. Protoplasts were analyzed immediately after isolation to obtain reliable results. Protoplasts suspended in cold W5 buffer were run through the cytometer at a medium flow rate. To stain the protoplasts, a final concentration of 2 μg/mL of FDA, 10 μg/mL Hoechst and 50 nM MitoTracker was used. The protoplasts were passed through a 488 nm laser to select for FDA-stained cells, 530/30 nm emission. FDA-positive protoplasts were gated and, Hoechst and MitoTracker stained protoplasts were quantified using the 405 nm excitation laser, 450/50 nm emission and 488 nm excitation, 585/42 nm emission, respectively.

The parameters simultaneously collected included linear forward scatter (FSC), linear side scatter (SSC), log FITC-A (for FDA dye), log BV510-A (for Hoechst dye) and log PE-A (for MitoTracker dye). At least 10,000 gated events of protoplasts were collected per sample, with three independent replicates. Protoplast populations were initially identified through the use of FSC and SSC. For subset gating, unstained controls were employed to identify a single distinct peak of protoplasts as the negative control for each of the wavelengths. Subsequently, the viability dye FDA was introduced to distinguish between viable and non-viable protoplasts. Within the population of viable protoplasts, those fluorescing with Hoechst were gated. Finally, within the subset of Hoechst-positive protoplasts, MitoTracker-positive protoplasts were identified.

### Regeneration of protoplasts in different mannitol concentrations

To 1 mL of protoplast solution, 4 mL of pea broth with varying mannitol concentrations (0, 0.1 M, 0.3 M, 0.5 M, 0.7 M and 0.9 M) was added, inverted once, and incubated at 24 °C. After 18–20 h, the tubes were centrifuged at 1000 g for 5 min and the supernatant was discarded. The pellet was resuspended, and protoplasts displaying visible hyphae were identified as regenerated protoplasts and were quantified using a hemocytometer. The regeneration rate was calculated as $\frac{number\ of\ regenerated\ protoplasts}{initial\ number\ of\ protoplasts} \times 100\mathrm{\%}$. The media that regenerated the highest number of protoplasts was termed as the regeneration medium for the remaining experiments. Images were captured with a light microscope (Zeiss, Göttingen, Germany). Images are representative of three biological replicates (with at least 20 protoplasts per replicate) for each treatment.

### Transformation of protoplasts derived from germinated cysts of *P. cinnamomi*

#### Bacterial plasmid with a cyan fluorescent protein gene insert

A plasmid (pCFPN) containing the cyan fluorescent protein gene (*CFP)* and neomycin phosphotransferase II (*nptII*) gene, as shown in Supplementary Fig. 1a, was provided by Prof. Howard Judelson, The Judelson Laboratory (University of California, Riverside, USA).

#### Electroporation of plasmids and extraction

Plasmid pCFPN was inserted into electrocompetent Mach1® *Escherichia coli* (provided by Soomin Lee, Deakin University, Australia) through the electroporation method described by New England BioLabs, Australia. Single colonies of the regenerated *E. coli* were transferred to Luria–Bertani (LB) liquid media with 100 μg/mL carbenicillin, and plasmid was extracted [QIAprep® Spin Midiprep Kit (Qiagen, Australia)] following the manufacturer’s instructions. Restriction enzymes *NheI* and *BsaI* (New England Biolabs, Australia) were used as preliminary validation to confirm the accuracy of the plasmid construct provided. Plasmid regions for *nptII* and *CFP* were amplified with oligos listed in Supplementary Table 1. The plasmid product was separated on a 1.5% (w/v) agarose gel (Supplementary Fig. 1).

#### Antibiotic growth trial

The antibiotic geneticin, also known as G418, interferes with protein synthesis and is commonly used as a selective agent during transformation of oomycetes and other eukaryotic cells (Evangelisti et al. 2019b).To determine the minimum concentration of G418 disulfate salt (Sigma-Aldrich, Australia) required to inhibit the growth of *P. cinnamomi,* mycelia and protoplasts were grown on 10% cV8 agar or pea broth agar, supplemented with varying G418 concentrations - 0 μg/mL (control), 10 μg/mL, 20 μg/mL, 30 μg/mL and 40 μg/mL. Plates were incubated at 24 °C in the dark and the growth was monitored for up to 14 days. The experiment was repeated thrice, with three plates per repeat.

For the mycelial growth on antibiotics, from a 3-day old *P. cinnamomi* plate, a 5 mm diameter plug was excised and placed on plates with different G418 concentrations. For the protoplast growth study, to 1 mL protoplast solution (10^5^–10^6^ protoplasts/mL concentration) in a 50 mL centrifuge tube, 2 mL of regeneration medium was added, inverted once, and incubated for 2 min. A further 15 mL of regeneration medium was added with carbenicillin at a final concentration of 50 μg/mL. The tube was then placed on its side at 24 °C for 20–22 h. The tube was then centrifuged at 1000 g for 5 min and the supernatant was discarded, leaving behind 2–3 mL liquid. The pellet was resuspended and 250 μL of the regenerated protoplast suspension was added to 10 mL of molten regeneration medium, with varying G418 concentrations.

#### PEG-mediated single plasmid transformation

The protocol for producing *P. cinnamomi* transformants was similar to a previously described method (Fang et al. 2017a), with modifications. Transformation was performed using 1 mL of protoplast in MMG solution (0.4 M mannitol, 15 mM MgCl_2_.6H_2_O, 4 mM 4-morpholinoethanesulfonic acid, pH 5.7) (1 × 10^5^–1 × 10^6^ protoplasts) with 40–50 mg of plasmid DNA (pCFPN) in a 50 mL centrifuge tube. The protoplast-plasmid solution was mixed completely by gently tapping the tube and then incubated on ice for 20 min. To each tube, 1.74 mL of freshly prepared PEG/calcium chloride (CaCl_2_) solution (40% w/v PEG 4000, 0.2 M mannitol, and 0.1 M CaCl_2_) was gradually added and gently mixed by rotating the tube. Then, after 15 min of incubation on ice, 2 mL of the regeneration medium was added, and the solution was mixed by gently inverting the tube, and it was placed on ice for a further 2 min. Then 3 mL of cold regeneration medium was added to the tube, mixed gently, and placed on ice for another 2 min. A further 10 mL of cold regeneration medium containing carbenicillin (final concentration 50 μg/mL) was then added to the tube. The tube was laid on its side and incubated overnight at 24 °C. After 20–22 h, the tube was centrifuged at 1000 g for 5 min and the supernatant was discarded, leaving behind 2–3 mL of liquid. The pellet was resuspended and 10 mL of regeneration medium with G418 (final concentration 20 μg/mL) was added, and the content was poured onto a 90 mm diameter Petri plate. The plates were incubated at 24 °C in the dark, until colonies appeared. On average, it took 7–10 days for colonies to emerge.

#### Screening of transformants

For screening of the transformants, individual colonies that appeared on the G418 plate were transferred onto 10% cV8 agar with 20 μg/mL G418 and incubated for 3–5 days in the dark. After two more rounds of screening, the regenerants were subjected to PCR verification for the presence of the *nptII* and *CFP* gene, using genomic DNA (gDNA) and complementary DNA (cDNA). gDNA was extracted using cetyltrimethylammonium bromide (CTAB)-based method (Islam et al. 2020) and PCR was conducted with MyTaq™ HS Red Mix 2x (Meridian Bioscience, Australia) following the supplier’s instructions. RNA was extracted using Isolate II RNA Plant Kit (Bioline, Australia) and cDNA was synthesized using SensiFast™ cDNA Synthesis Kit (Bioline, Australia). The primers used are listed in Supplementary Table 1. PCR products were visualized using 1.5% agarose gel electrophoresis. Each set of reactions included samples of wildtype (Pc-WT) as the negative control and the plasmid, pCFPN, as the positive control. Screening using PCR was performed in triplicates to validate the results. Overall transformation efficiency was calculated using the formula: $\frac{number\ of\ positive\ transformants}{total\ number\ of\ regenerants} \times 100\mathrm{\%}$  

#### In vitro growth and *in planta *virulence activity of the transformants

For in vitro growth and *in planta* study, one transformant per replicate was selected for comparative study. To compare the growth rate in vitro, plugs were taken from a 3-day old *P. cinnamomi* plate of Pc-WT and the transformants were subcultured onto 10% cV8 agar, in the absence or presence of G418. Images of the colony morphology were captured, and the growth area was measured using ImageJ software. Three repeat plates were used for each isolate and the experiment was performed in three replicates.

For *in planta* analysis, plugs of Pc-WT and the transformants were used to inoculate lupin roots grown in a soil-free system (Allardyce et al. 2012). Lesions formed were measured on days 1,3,5 and 7 after inoculation. For negative control, sterile agar plugs excised from 10% cV8 agar was used. Images of the root were captured daily (up to Day 7), and the root length and lesion length was measured using ImageJ software. The experiment was repeated thrice, with 8 plants per replicate.

## Results

### Validation of fluorescent dyes for use in *P. cinnamomi*

FDA is a non-fluorescent molecule that readily permeates the cell membrane and, upon intracellular esterase activity, is converted into the green fluorescent molecule fluorescein. This transformation enables the visualization of viable and intact cells, distinguishing them from non-viable cells that do not fluoresce (Johnson et al. 2013). Hoechst is a membrane permeable stain that binds to the adenine–thymine regions of the minor grooves of the DNA (Chazotte 2011). MitoTracker is a fluorescent dye that irreversibly binds to the polarized mitochondrial membrane, (Poot et al. 1996) and specifically stains live cell mitochondria depending on the organelle’s membrane potential, making it a widely utilized tool in research related to human health (Xu et al. 2021; Xiao et al. 2016).

**Fig. 1:**
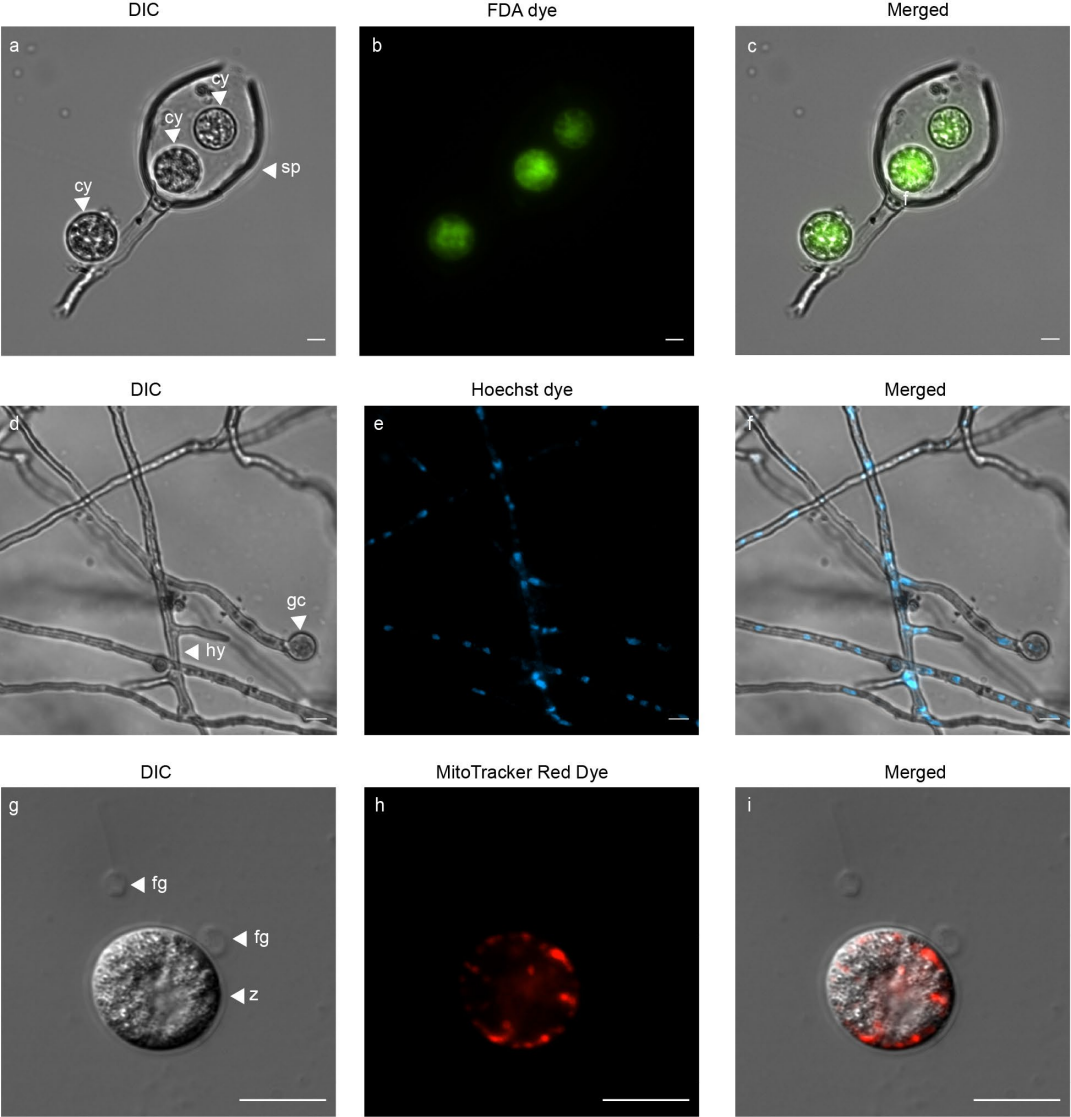
Light microscope images of *P. cinnamomi* stained with fluorescent dyes **a** *P. cinnamomi *sporangia (sp) with encysted zoospores (cy). **b** Each cyst is emitting green fluorescence when stained with FDA. **d** Germinated cyst (gc) and hyphae (hy) of wild type *P. cinnamomi *(Pc-WT), with DIC microscopy. **e** Nuclei within the hyphae is distributed throughout the coenocytic hyphae, stained with Hoechst 33342 (blue). **g** Zoospore at the point of encysting (z), with the flagella (fg) detached. **h** The mitochondria, stained in red with MitoTracker™ Red CMXRos, is spread along the periphery of the encysting zoospore, underlying the cell membrane. **c**, **f** and **i** are images with the individual channels merged. (Scale = 10 μm)

As an initial step in the validation of viability and organelle specific fluorescent dyes, sporangia, zoospores and germinated cysts of Pc-WT were prepared. DIC microscopy coupled with fluorescence microscopy (Fig. 1) was used to validate both viability and location of nuclei and mitochondria. Sporangia with fluorescing cysts (Fig. 1a-c) was achieved with FDA stain. Overnight germination of cysts resulted in elongated hyphae (Fig. 1d) and Hoechst staining revealed the distribution of nuclei within the hyphae (Fig. 1e). A single zoospore with detached flagella is shown in Fig. 1g using DIC microscopy, and MitoTracker selectively stained mitochondria within the encysting zoospore (Fig. 1h). The merged images depict the dispersion of nuclei within coenocytic hyphae (Fig. 1f) and the distribution of mitochondria along the periphery of encysting zoospores, positioned beneath the plasma membrane (Fig. 1i).

### Visualization of organelle-specific fluorescent dyes in *P. cinnamomi*

**Fig. 2:**
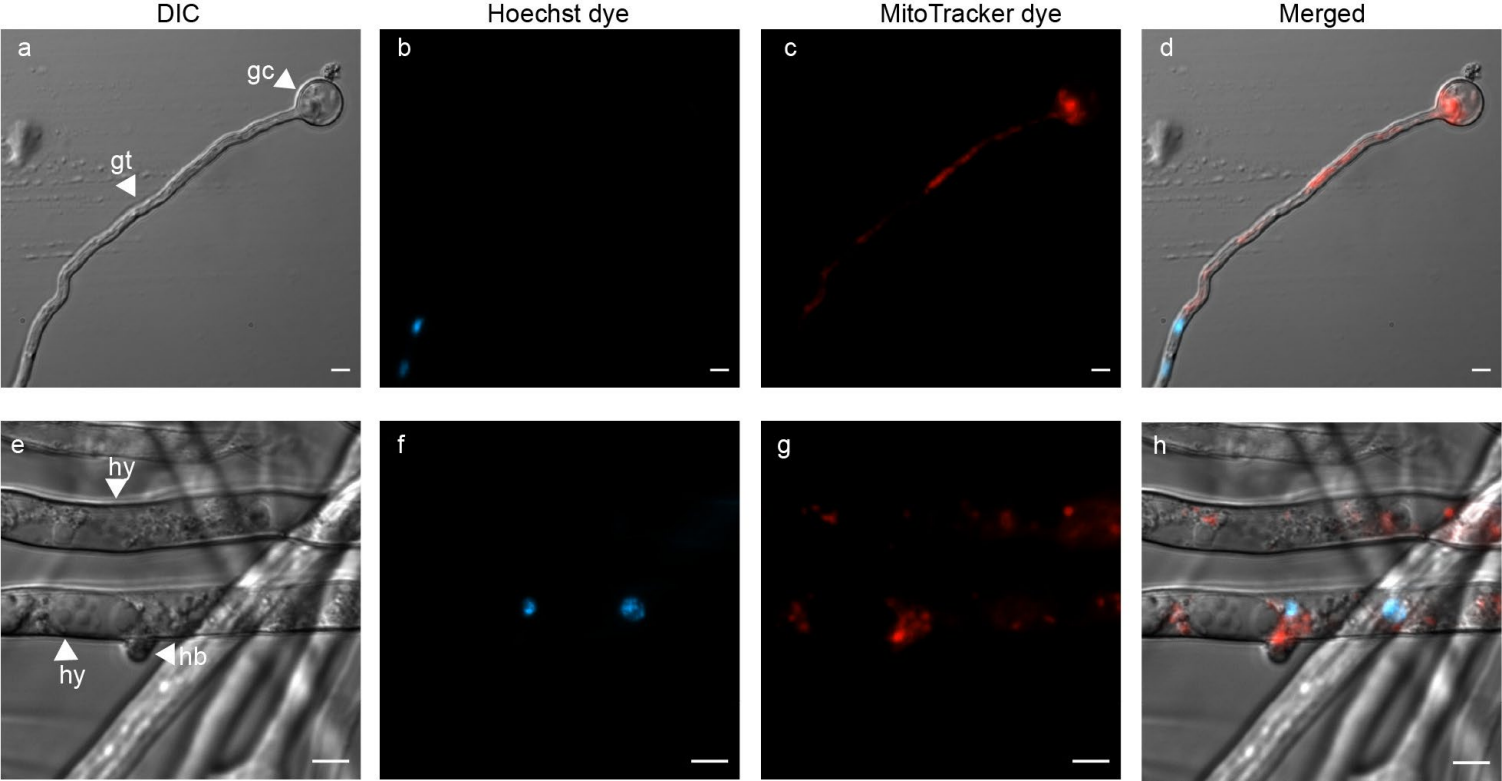
Use of dual fluorescent dyes to visualize organelles in asexual structures of *P. cinnamomi* **a** Germinated cyst (gc), with a growing germ tube (gt). **b** and **c** germ tube harbouring two nuclei (blue) and multiple mitochondria (red), respectively **e** A high resolution image that shows mature hyphae (hy), with hyphal branching (hb). **f** and **g** shows two nuclei and multiple mitochondria spread along the cytoplasm of the hyphae, respectively. **d** and **h** are images with the individual channels merged. (Scale = 5 μm)

In Fig. 2, we demonstrate the dual staining capability of Hoechst and MitoTracker for nucleus and mitochondrial visualization, respectively. A germinated cyst with a growing germ tube (Fig. 2a) has two nuclei stained in blue (Fig. 2b), and mitochondria spread across the germ tube, stained in red (Fig. 2c). In the high-resolution image of Pc-WT hyphae (Fig. 2e), two nuclei are present in only one of the hyphal segments (Fig. 2f) while mitochondria are distributed (Fig. 2g) throughout the hyphae. The merged images (Fig. 2d and Fig. 2h) showcase the precise localisation of the nucleus and mitochondria within the germ tube and hyphae, respectively.

### Protoplast isolation and regeneration

The quality of protoplast formation is critical, as it varies depending on the age and developmental stage of the oomycete, thereby impacting transformation efficiencies. Additionally, factors such as regeneration medium, osmotic stabilizers, enzyme type and concentration and digestion duration are essential for the preparation of healthy and viable protoplasts. Previous research had optimized enzymatic digestion parameters for *P. cinnamomi* (Dai et al. 2021). In our study, we concentrated on optimizing the starting material used, growth media and regeneration media to generate healthy protoplasts suitable for subsequent transformation.

#### Effect of starting material and growth media on protoplast isolation

Protoplasts isolated from *P. cinnamomi* were spherical, with size ranging from 10 – 35 μm. In this study, the highest protoplast yield was obtained from the culture in the exponential phase of growth, germinated cysts (i.e. encysted zoospores incubated for 18 h). The germinated cysts formed a thin mat (Supplementary Fig. 2) and were completely digested to release protoplasts, with concentrations ranging from 3.7 × 10^6^–8.2 × 10^6^ protoplasts/mL, depending on the type of media used. Incubation of encysted zoospores in growth media for a shorter time resulted in less starting material to work with. Incubation for longer than 18 h generated a thick mat that attached to the sides of the tube and were difficult to harvest. Instead of germinated cysts, when mycelial plugs were used as the starting material, incubation for 40 h or longer resulted in incomplete enzymatic digestion. This highlights the variability in the growth rate of different isolates of *P. cinnamomi*, as evidenced by the discrepancy between our findings and those of a prior study (Dai et al. 2021), which utilized a 2.5-day-old mycelial mat for enzymatic digestion.

**Fig. 3:**
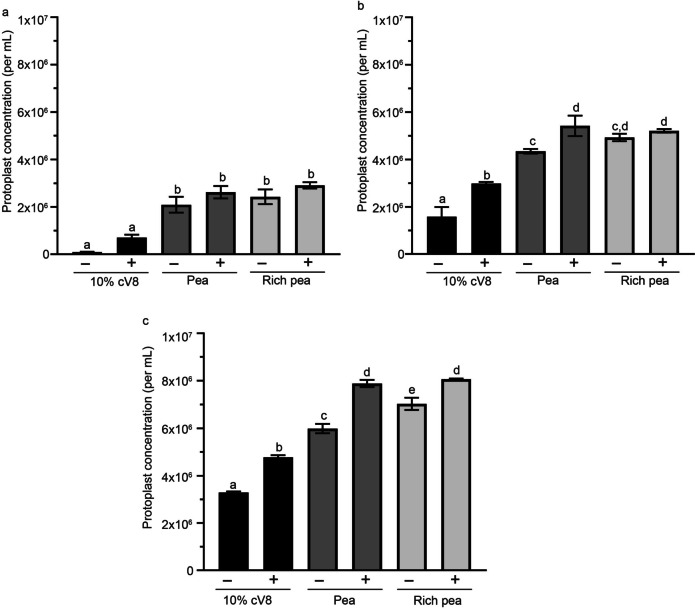
Comparison of the effect of starting material and growth media on *P. cinnamomi *protoplast yields. Three different growth media (10% cV8, pea and rich pea), without (-) or with (+) β-sitosterol, were used for the growth of the starting material in order to isolate protoplasts. The starting material used was: **a** Mycelia harvested from plugs incubated in media for 18 hours. **b** Mycelia harvested from plugs incubated in media for 40 hours. **c** Germinated cysts harvested from media after 18 hours of incubation. Different letters above each bar diagram represents a significant difference calculated using 2-way ANOVA and Tukey multiple comparison

As shown in Fig. 3, the starting material of *P. cinnamomi* and the media used for culturing it, both had a significant effect on the number of protoplasts isolated. Use of the most common media for *P. cinnamomi* growth and maintenance, 10% cV8 broth, generated the least number of protoplasts, regardless of the starting material used. Across all media types, the addition of β-sitosterol consistently promoted isolation of a higher number of protoplasts.

Protoplast liberation was highest and comparable for starting material grown in pea broth and rich pea broth, both supplemented with β-sitosterol. Hence, unlike *P. sojae* whereby rich pea broth is used for protoplast isolation (Fang et al. 2017a), a cheaper and quicker alternative media of pea broth supplemented with β-sitosterol is sufficient for protoplast isolation from *P. cinnamomi.*

Enzyme digestion using cellulase and lysing enzyme for 45 min yielded the best protoplast concentration, even though some starting material may remain undigested. We found that enzyme digestion for more than 90 min generated large, vacuolated, and unhealthy protoplasts.

#### Effect of osmoticum on protoplast regeneration

In a previous study by Dai et al. (2021), mannitol-free media was used for regeneration of protoplasts followed by addition of agar with 0.5 M mannitol after 20 h of incubation. In our study, we have presented quantitative data alongside microscope images to demonstrate the importance of mannitol during the initial stages of protoplast regeneration. Through a comparison of protoplast regeneration in varying mannitol concentrations, we found that 0.5 M mannitol yielded the highest rate of protoplast regeneration, leading us to term this solution as the regeneration medium.

**Fig. 4:**
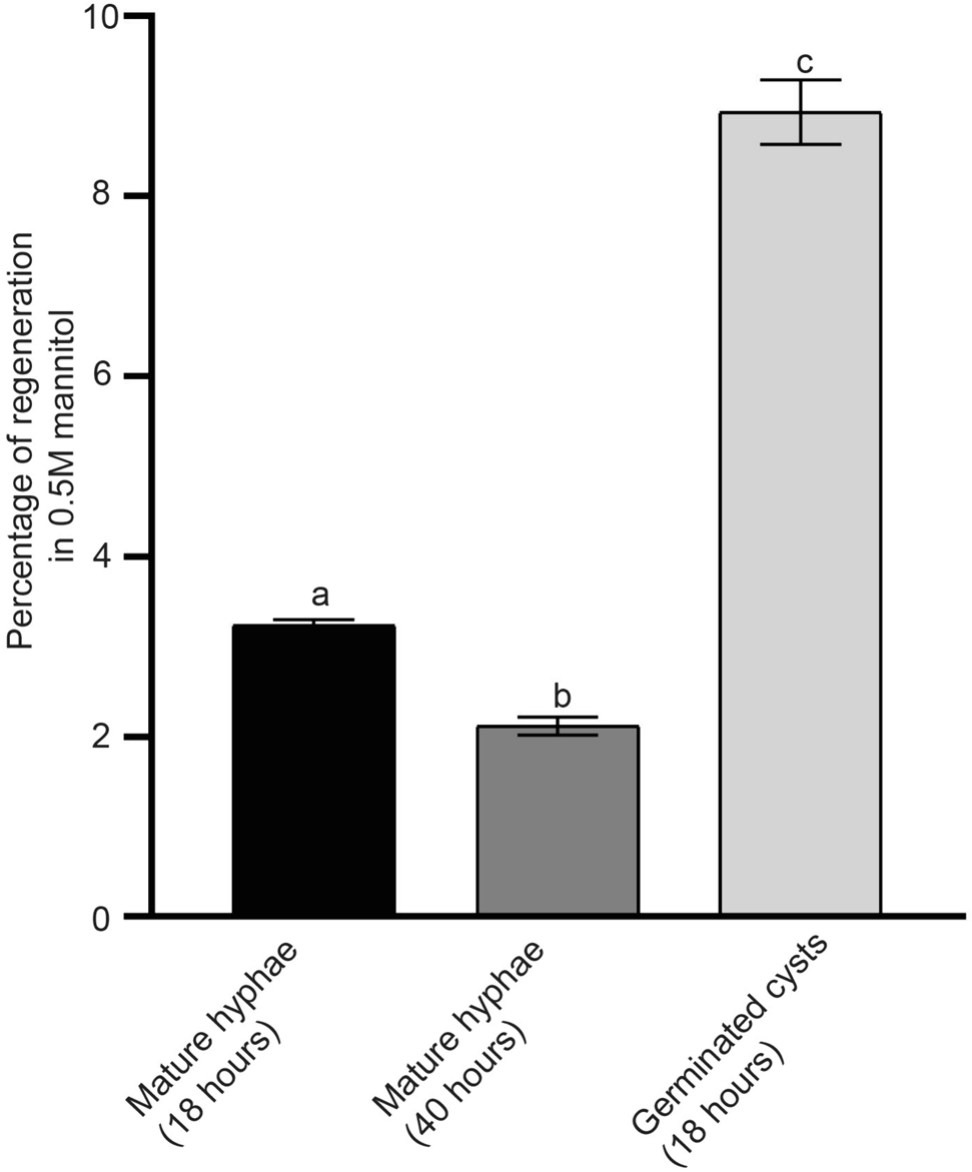
Summary of percentage of regeneration of protoplasts in pea broth. Protoplasts isolated from different starting materials (mature hyphae of 18 or 40 hours, or germinated cysts of 18 hours) grown in pea broth with β-sitosterol were regenerated in pea broth with 0.5 M mannitol. Evidently, protoplasts generated from germinated cysts had the highest percentage of regeneration. Different letters above the error bars indicate significant difference calculated using 2-way ANOVA and Tukey multiple comparison

As shown in Fig. 4, protoplasts isolated from germinated cysts in regeneration medium had the highest rate of recovery at 8.93%. Even though mycelia generated from plugs incubated for 40 h generated a high number of protoplasts, the percentage of regeneration was the least, at 2.12%. This low percentage viability indicated that most protoplasts yielded from these hyphae were vacuolated and non-viable.

The regeneration of protoplasts was greatly inhibited in the media which lacked mannitol and in media with mannitol concentrations above 0.5 M (Supplementary Fig. 3). Morphological differences were observed in protoplasts that regenerated in varying mannitol concentrations (Supplementary Fig. 4). At 0.5 M mannitol concentration, the regenerated protoplasts had elongated, uniform hyphae. At 0.7 M and 0.9 M mannitol concentrations, short and swollen hyphae were observed.

### Use of vital stains to confirm protoplast integrity and viability

**Fig. 5:**
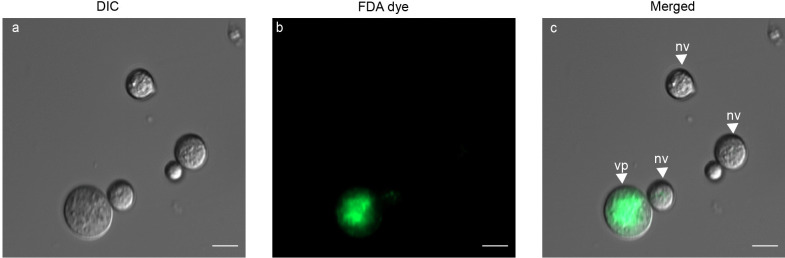
Fluorescein diacetate (FDA)-stained protoplasts of *P. cinnamomi*. **a** Protoplasts, of varying sizes, with DIC microscopy. **b** Protoplast emitting bright, green fluorescence suggesting the protoplast is intact and contains fluorescein esterase within the cell (viable). Protoplasts that lack the fluorescence are non-viable. **c **Image with the individual channels merged. Viable protoplasts are labelled vp, while non-viable protoplasts are labelled as nv (Scale = 10 μm)

**Fig. 6:**
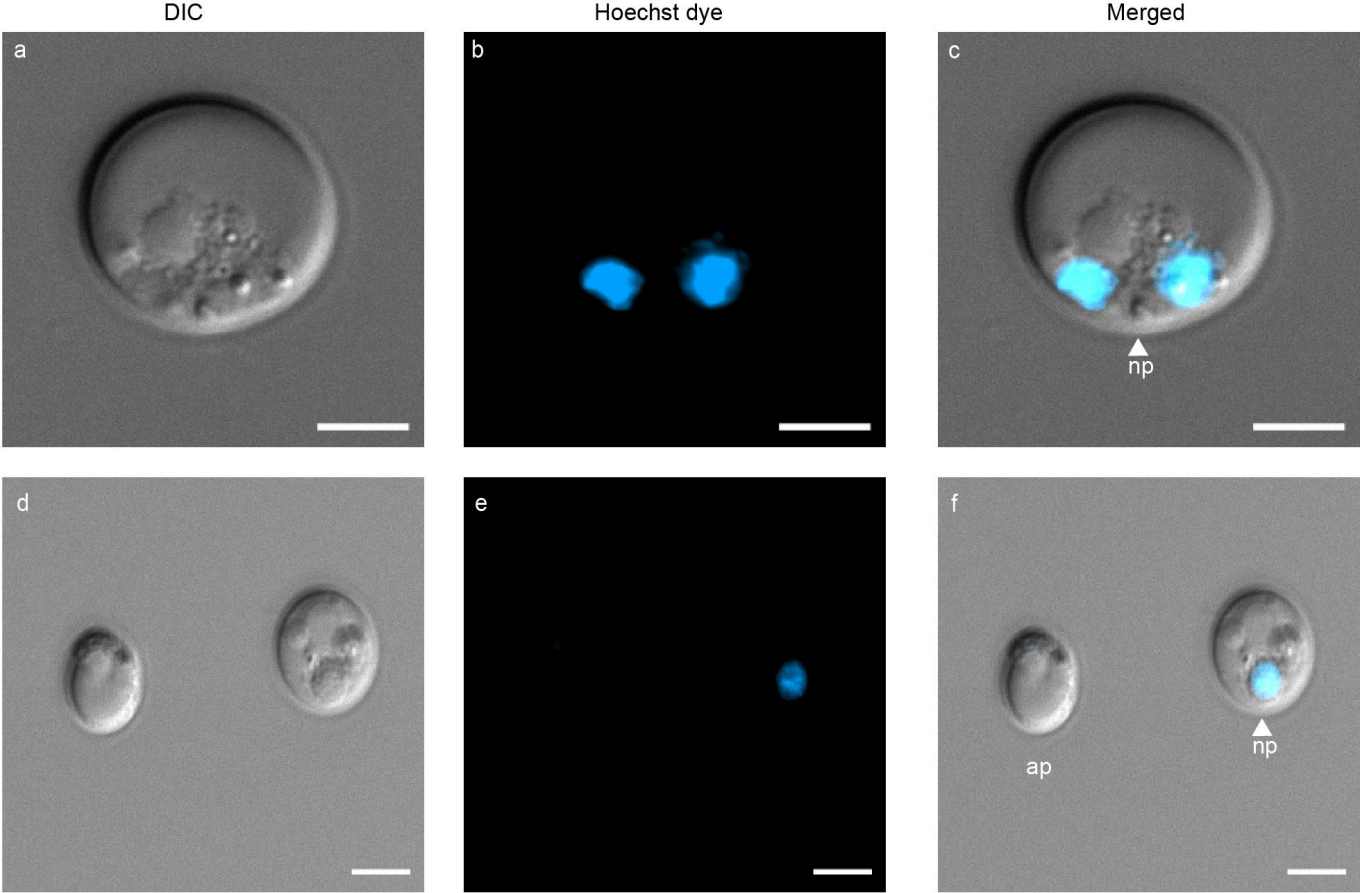
Hoechst 33342 stained nuclei of *P. cinnamomi *protoplasts **a** A high-resolution image of protoplast with DIC microscopy. **b** Two nuclei within the protoplast visualized with Hoechst 33342 stain **d** Protoplasts of varying sizes. **e** Only one of the protoplasts contains a nucleus, while the other protoplast is anucleated. **c** and **f** are images with the individual channels merged. Protoplasts with nucleus (np) and without (anucleated protoplast - ap) are labelled in the image. (Scale = 5 μm)

**Fig. 7:**
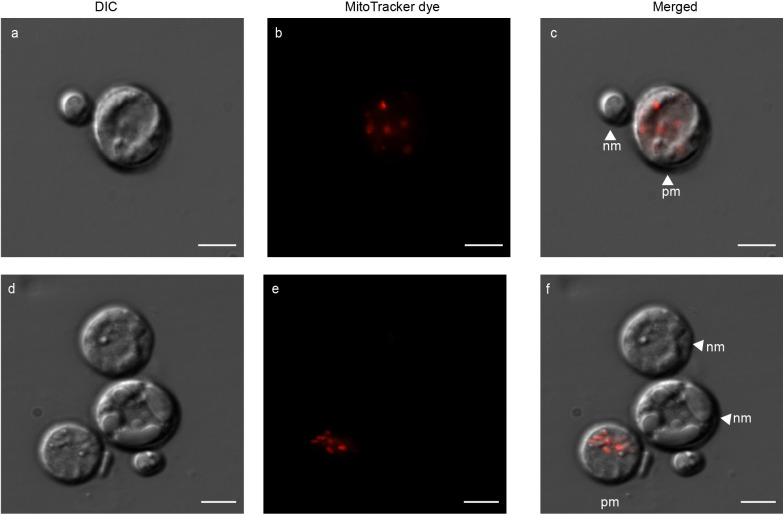
Novel use of MitoTracker™ Red CMXRos to stain mitochondria within *P. *cinnamomi protoplasts **a** Protoplasts of varying sizes with DIC microscopy. **b** Mitochondria present within one of the protoplasts is visible, with MitoTracker™ Red CMXRos stain. **d** Multiple protoplasts clustered together. **e** Only one of the protoplasts harbours mitochondria, while the others have non-functional mitochondria or lack them completely. The mitochondria are randomly spread within the protoplast. **c** and **f** are images with the individual channels merged. Indicated are the protoplasts with mitochondria (pm) and protoplasts which either lack mitochondria or, where present, are non-functional (nm). (Scale = 5 μm)

The use of FDA resulted in a mixed population of protoplasts that emitted strong fluorescence versus no fluorescence, confirming that FDA selectively stained for live cells. Protoplasts of different sizes are shown in Fig. 5a, with FDA-stained protoplasts fluorescing green (Fig. 5b). Protoplasts that did not fluorescence were non-viable. In Fig. 5c, merged image is presented, with the viable protoplasts labelled. Visualization of Hoechst-stained protoplasts confirmed these cells contained varying numbers of nuclei (Fig. 6). A high-resolution image of a single protoplast is presented in Fig. 6a, with the nuclei emitting blue light when stained with Hoechst (Fig. 6b). The merged image shows the bi-nucleated protoplast, with cytoplasmic content within (Fig. 6c). In Fig. 6d, two protoplasts were visualized with DIC microscopy, followed by Hoechst stain to visualize the nucleus (Fig. 6e). The merged image (Fig. 6f) identified one protoplast as uninucleated, while the other exhibited an absence of nuclear content. Throughout the imaging process, we observed protoplasts with as many as three nuclei. The application of MitoTracker to label the mitochondria within protoplasts proved that some protoplasts contained mitochondria, while others lacked them entirely or had non-functional mitochondria (possibly due to insufficient membrane potential) (Fig. 7). Protoplasts of varying sizes were visualized with DIC microscopy (Fig. 7a and Fig. 7d). In both Fig. 7b and Fig. 7e, the protoplasts exhibiting red fluorescence signify the binding of MitoTracker stain to the mitochondria. The merged images, Fig. 7c and Fig. 7f confirms the placement of the mitochondria within the protoplast.

**Fig. 8:**
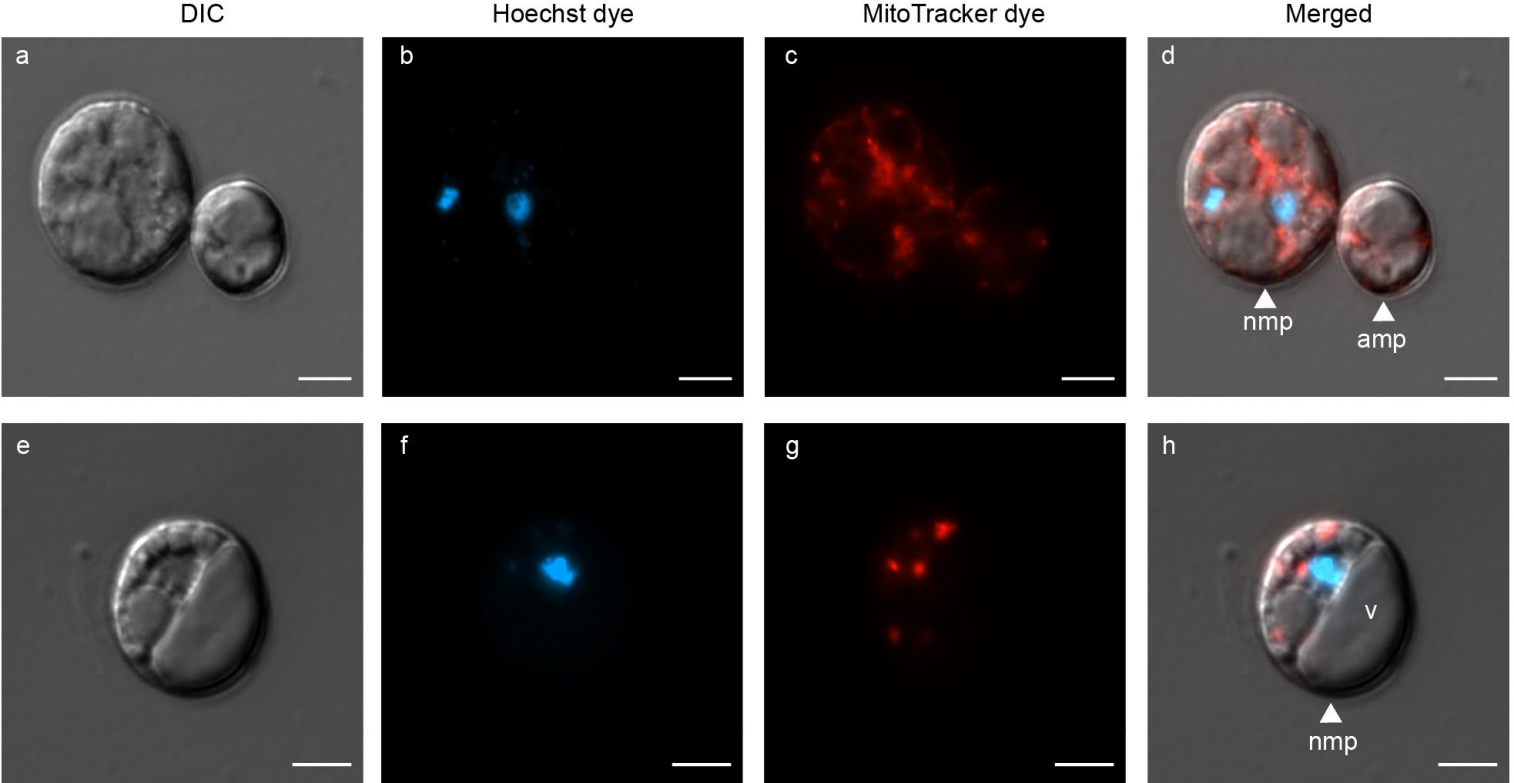
Dual staining of *P. cinnamomi *protoplasts to visualize the nucleus and mitochondria **a **and **e **High resolution image of protoplasts, with DIC microscopy. **b** Hoechst 33342 stained two nuclei present within one of the protoplasts, while the other protoplast is anucleated. **c** Mitochondria within the protoplasts are fluorescing red as they were stained with MitoTracker™ Red CMXRos. **f** and **g** Protoplast with a single nucleus and multiple mitochondria, respectively. **d** and **h** are images with the individual channels merged. Indicated are protoplasts that contain both nuclei and mitochondria (nmp), and an anucleated protoplast with mitochondria (amp). The vacuole **(v)** is labelled as well. (Scale = 5 μm)

Through dual staining (Fig. 8) with Hoechst and MitoTracker, we observed differences in the cellular composition of protoplasts, in terms of nuclei and mitochondria. In Fig. 8a and Fig. 8e, protoplasts of varying sizes were visualized with DIC microscopy. In Fig. 8b two nuclei were present within the protoplast, while in Fig. 8f the protoplast had only one nucleus. Furthermore, all protoplasts visualized in Fig. 8c and Fig. 8g contained mitochondria. The merged image in Fig. 8d and Fig. 8h demonstrates that in *P. cinnamomi* protoplasts, the presence of a nucleus does not necessitate the presence of functional mitochondria, and vice-versa. Thus, *P. cinnamomi* protoplasts can exhibit a diverse cellular composition, but for successful regeneration, they require all the cell organelles. Overall, protoplasts lacking functional cell organelles were deemed non-viable and unhealthy.

#### Application of flow cytometry to differentiate protoplast populations

**Fig. 9:**
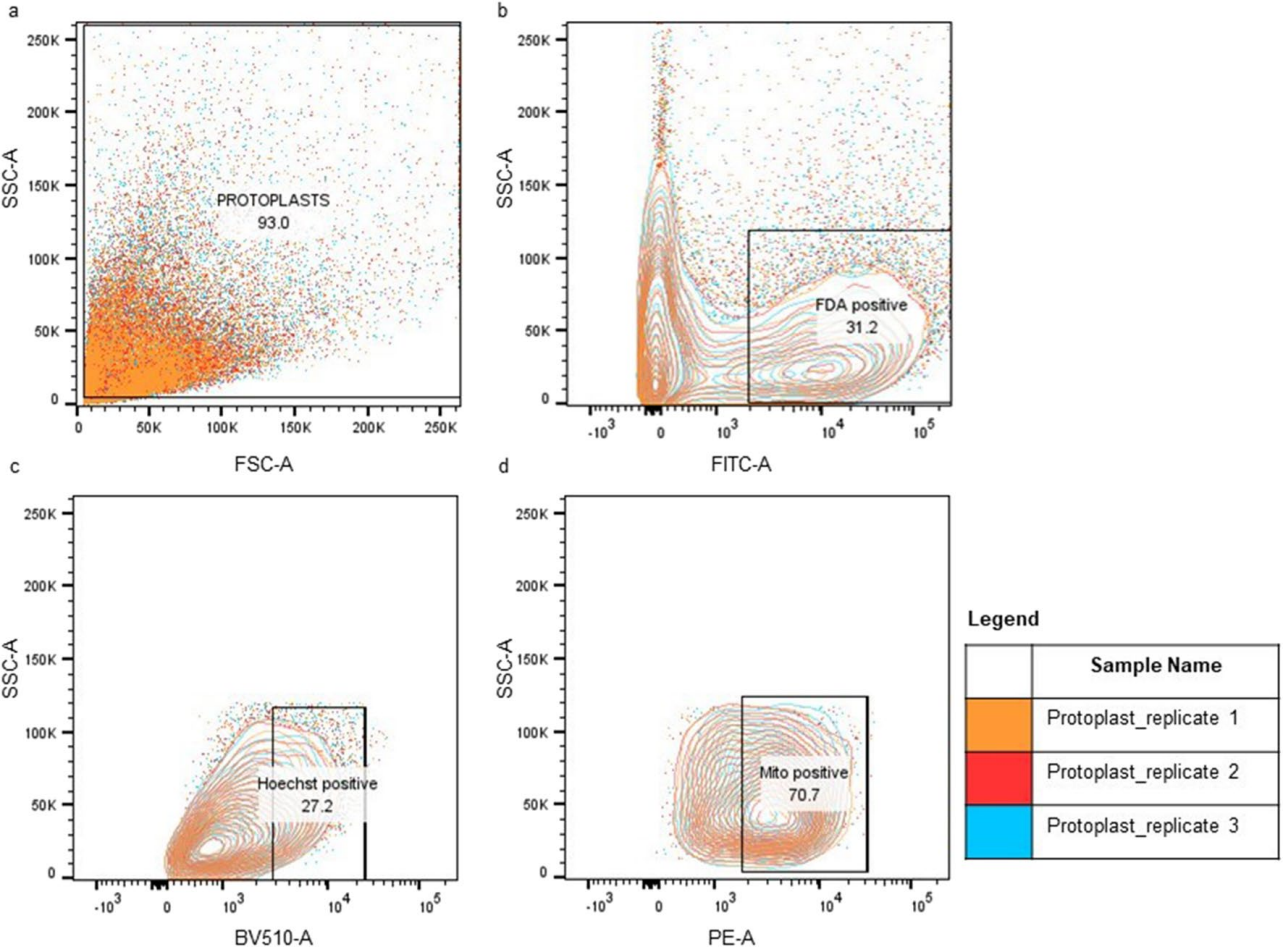
Flow cytometric analysis of the protoplasts isolated from germinated cysts (18 hours) of* P. cinnamomi.* **a** Approximately 93% of events were gated for protoplast. The total number of protoplasts analyzed was 10,000. Given that *P. cinnamomi *protoplasts had a wide variety of sizes, almost all data points obtained were gated with the forward scatter channel (FSC-A) and the side scatter channel (SSC-A). **b** 31.2% of FDA positive protoplasts were identified with the FITC-A channel, suggesting that in this protoplast population only 3120 protoplasts were viable. **c** From the viable protoplast population, protoplasts with at least one nucleus were identified with Hoechst 33342 stain, under BV510-A channel (27.2%). **d** Within the Hoechst 33342 positive population, protoplasts containing mitochondria were identified with MitoTracker™ Red CMXRos stain, under PE-A channel (70.7%)

A FCM dot plot of *P. cinnamomi* protoplasts isolated from germinated cysts grown in pea broth with β-sitosterol was obtained, as shown in Fig. 9. All data points for protoplasts were gated (Fig. 9a) and a clear distinction of protoplast populations stained with and without the FDA stain is visible (Fig. 9b), with the FDA-positive cells (live protoplasts) representing 31.2% of the overall protoplast population. Cells gated for positive FDA stain were then subjected to Hoechst stain selection that identified protoplasts containing at least one nucleus. Figure 9c shows that from the live protoplast population, only 27.2% of protoplasts contained at least one nucleus. Following that, Hoechst positive protoplasts were subject to MitoTracker stain (Fig. 9d) and it was found that the majority of the protoplasts that contained a nucleus had functional mitochondria as well, at 70.7%.

Through microscopy and FCM results, we confirmed a mixed population of protoplasts generated from *P. cinnamomi.* Each protoplast appeared distinctive, containing different numbers of nuclei, mitochondria and potentially, other cell organelles.

### Growth of Pc-WT in Geneticin

Interestingly, the inhibitory concentration of G418 on Pc-WT varied depending on the type of starting material and media used. When plugs from the growing edge of the plate were used as a starting inoculum, the minimum inhibitory concentration (MIC) of G418 on *P. cinnamomi* growth was determined to be 30 μg/mL in 10% cV8 agar and 40 μg/mL in pea agar. In contrast, protoplasts were highly sensitive to the presence of G418 and concentration of the antibiotic above 20 μg/mL inhibited the regeneration process (Supplementary Fig. 5).

Morphological differences were present in Pc-WT cultures grown in media supplemented with G418. In the presence of G418, the hyphae produced more coralloid structures and were highly branched. At 30 μg/mL and 40 μg/mL G418 concentrations, the cytoplasmic content had irregular distribution within the hyphae, with eventual death of the mycelia over an extended incubation period. In contrast to a study that reported 70 μg/mL of G418 was required to inhibit growth of wildtype *P. cinnamomi* (Dai et al. 2021), our study demonstrated that at concentrations higher than 20 μg/mL, the pathogen growth was largely inhibited. This suggests the genetic differences present within different isolates of *P. cinnamomi*, that may depend on the region of isolation and the host plant it is associated with.

### Transformation of *P. cinnamomi*

#### Selection of transformants

We initially examined protoplast regeneration in plates supplemented with 30 μg/mL of G418 to ensure no wildtype regenerants would survive. However, this resulted in a complete loss of the protoplast regenerants, suggesting the conditions were too harsh for regeneration to occur.

A total of 46 G418-resistant putative transformants were regenerated, from the three replicates of protoplast transformation study, in G418 selective media screening (at 20 μg/mL). The regenerants were subjected to a rating based on their radial growth (Supplementary Fig. 6a-b). After subsequent subculturing, three transformants per replicate (total of 9, i.e. 19.5%) grew on the plate supplemented with G418, while the remaining did not grow. One regenerant from each replicate was randomly chosen for the remaining experiments and were named Pc-CFP-1, Pc-CFP-2 and Pc-CFP-4.

**Fig. 10:**
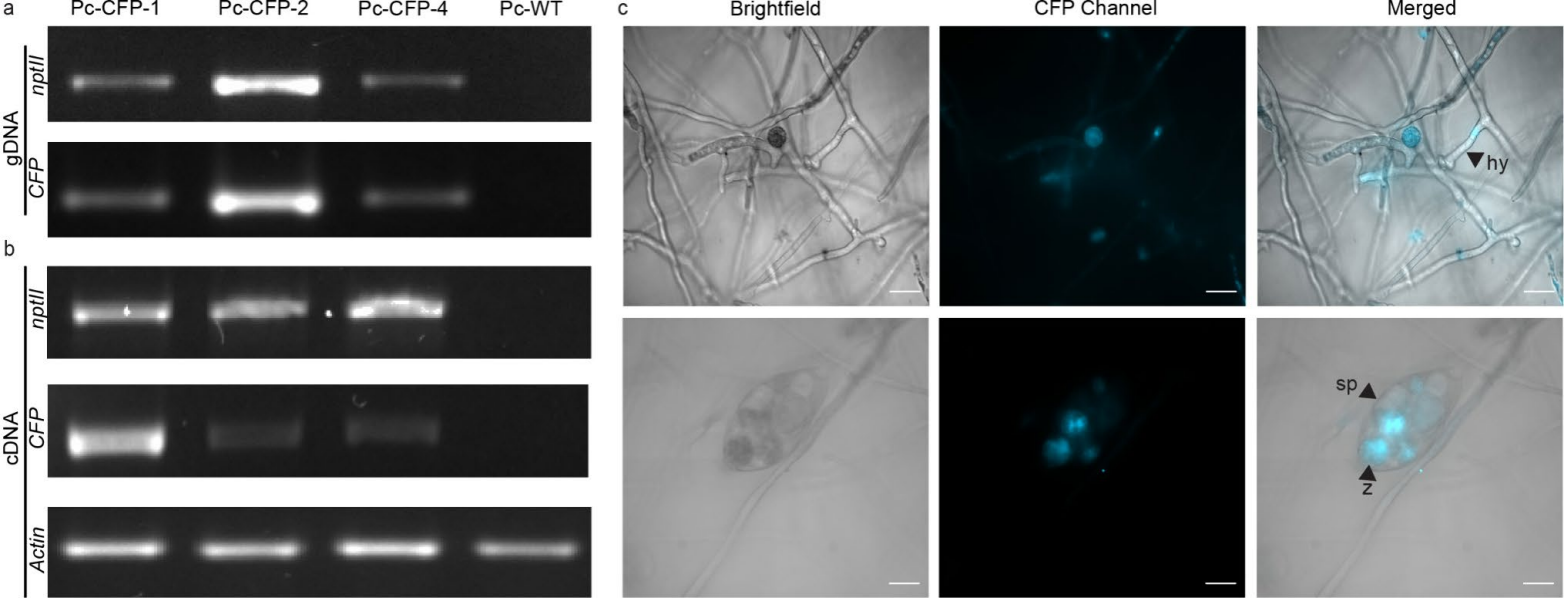
Validation of *P. cinnamomi *transformation through PCR and microscopy **a** and **b** PCR assay of *nptII *and *CFP* gene in G418-resistant transformants with genomic DNA (gDNA) and complementary DNA (cDNA), respectively. *P. cinnamomi *transformants, Pc-CFP-1, Pc-CFP-2 and Pc-CFP-4, contained the *nptII *and *CFP *gene. A faint band for *CFP *gene in cDNA for Pc-CFP-2 and Pc-CFP-4 suggests low expression of the fluorescent gene. Wildtype, Pc-WT, does not contain the *nptII *and *CFP *gene. Actin was used as an internal control for *P. cinnamomi. ***c **Microscopic visualization of the cyan fluorescent protein localised within the hyphae (hy) and zoospores (z) present within the sporangia (sp) of *P. cinnamomi *transformants. (Scale = 20 μm)

As shown in Fig. 10 (a-b), bands of 493 bp and 312 bp, for *nptII* and *CFP* respectively, were amplified from the gDNA and cDNA extracted from the three transformants, confirming the successful transformation of those cultures. No bands were amplified from the negative control, Pc-WT, for *nptII* and *CFP*. *Actin* was used as an internal control for *P. cinnamomi* and a band of 112 bp was obtained for all samples, except the plasmid. Microscopic analyses were carried out to examine the expression of the visual marker, *CFP* in various asexual structures, including the hyphae and zoospores of the transformants (Fig. 10c). Following a two-month interval, we conducted qPCR analysis on the transformants to evaluate whether the transformation resulted in transient or stable expression of *nptII* and *CFP* genes. The expression analysis (data not provided) indicated continued expression of the genes, confirming stable integration of the plasmid into the genome of *P. cinnamomi*.

#### Vegetative growth and virulence of Pc-CFP

**Fig. 11:**
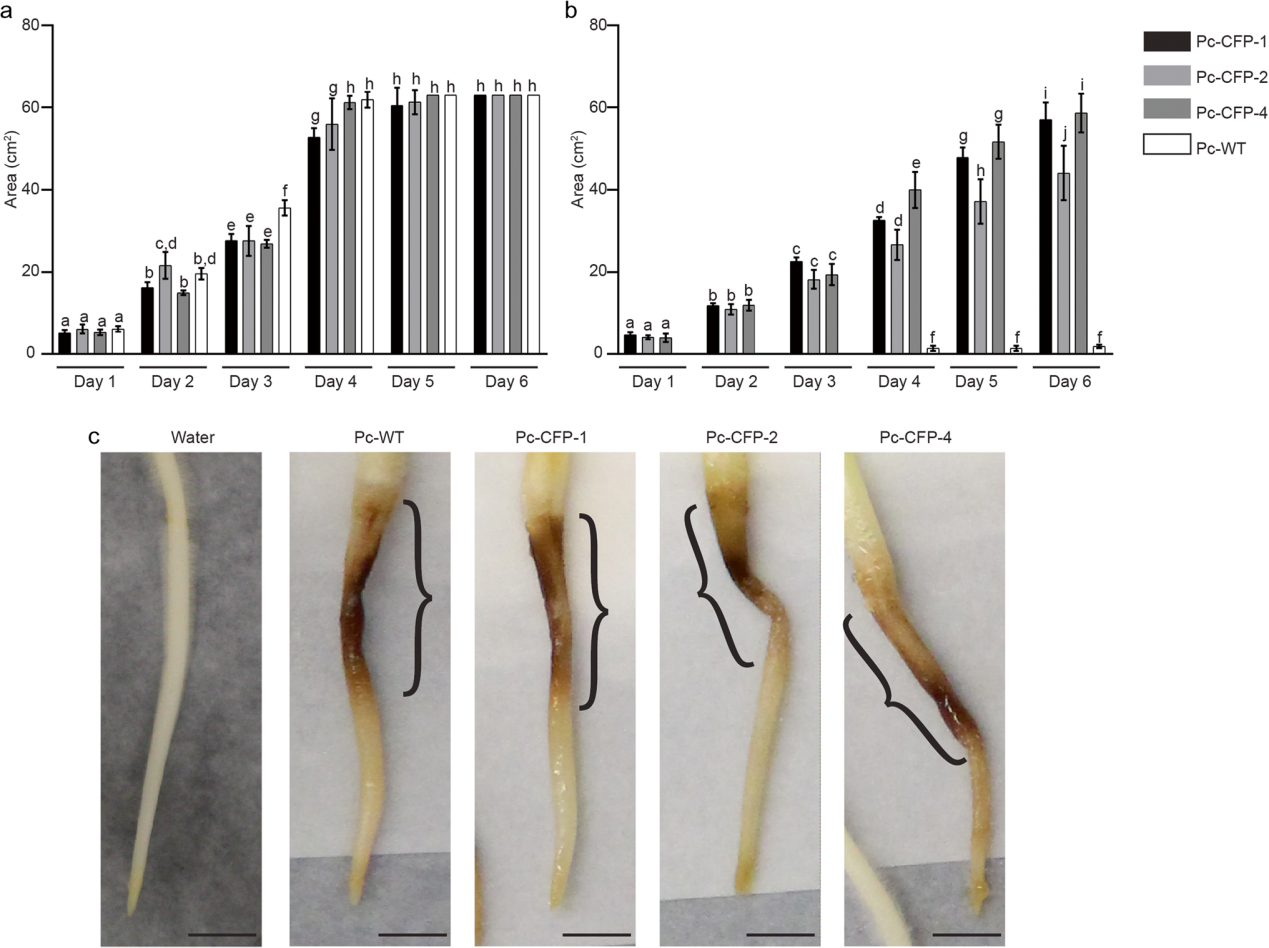
*in vitro *comparison of growth of *P. cinnamomi *transformants and wildtype, and *in planta *development of lesions in roots inoculated with the wildtype or transformants **a** and **b** compares the growth area of the mycelia of the transformants and wildtype in the absence and presence of G418, respectively. The x-axis represents days elapsed since the transfer of plugs. Different letters above each bar diagram represents a significant difference calculated using 2-way ANOVA and Tukey multiple comparison. **c** Lupin roots showing visible lesions (black brackets) developed following inoculation with wildtype *P. cinnamomi*, Pc-WT, and the transformants, Pc-CFP-1, Pc-CFP-2 and Pc-CFP-4, 7 days after inoculation. (Scale = 1 cm)

None of the transformants showed any abnormal mycelial morphology under microscopic examination when grown on 10% cV8 agar. By day 5, all transformants had an in vitro vegetative growth rate comparable to that of the wildtype, when grown without antibiotics (Fig. 11a, Supplementary Fig. 6c). For two of the transformants (Pc-CFP-1 and Pc-CFP-4) grown on antibiotic agar (Fig. 11b), their growth was similar to the wildtype (Pc-WT) grown without antibiotics. However, for one isolate, Pc-CFP-2, at Day 6, the growth was approximately 13.5% less, compared with that of the other two transformants. For Pc-WT, there was negligible growth observed in agar with antibiotics. When lupin roots were inoculated with either Pc-WT or one of the transformants (Fig. 11c), roots ceased growing and the length remained similar, as did the development of lesions.

## Discussion

In the present study, we have developed and optimized a protocol for production of protoplasts from germinated cysts of *P. cinnamomi*. We have shown that viability and organelle-specific stains are an addition to the toolbox to visualize and understand the composition of the protoplasts. We have also used flow cytometry (FCM), a powerful tool that has been traditionally used in animal and plant cell biology, for quantifying protoplast viability and organelle presence, such as the nucleus and mitochondria. Following the successful production of protoplasts, we have then used them in a robust transformation system in *P. cinnamomi* to generate transformants expressing cyan fluorescent protein.

### Insights into the cellular composition of *P. cinnamomi* – a fluorescence-based approach

Fluorescence-based imaging has transformed the field of cell biology by enabling visualization of the precise localisation of components within specific cells and organelles (Nadiminti et al. 2015; Frigault et al. 2009). In *Phytophthora* species, our knowledge of cellular biology has primarily relied on ultrastructural electron microscopy studies conducted in the past (Hardham 1987; Hemmes and Wong 1975). In recent years, Shetty et al. (2019) has used fluorescent dyes in oomycetes to monitor pathogen growth and development (Fairhurst et al. 2023). In our study, we showcase the application of three different fluorescent dyes to advance our comprehension of *P. cinnamomi* cell biology by initially staining asexual structures of the pathogen followed by protoplast stains, and have integrated this approach with FCM.

FDA is a widely utilized dye to assess cell viability and is commonly used in bacterial (Schumacher et al. 2015), human (Kanade et al. 2016) and plant studies (Pasternak et al. 2021). In oomycetes, this stain has been recently used to validate the viability of *P. agathidicida* oospores (Fairhurst et al. 2023). In our study, we corroborate the effectiveness of FDA to stain encysted zoospores of *P. cinnamomi*. When we generated a fresh batch of zoospores under identical conditions, all the zoospores germinated to develop into mature hyphae. This correlation supports the notion that FDA effectively stains viable zoospores.

Contrary to a previous study (Fang et al. 2017b) where Hoechst stain was ineffective in staining *P. sojae* hyphal nuclei, the stain effectively bound to the nucleus of *P. cinnamomi* and fluoresced. According to Boevink et al. (2020), the factors that determine the movement of cell organelles and nuclear materials within the hyphae remains unclear. However, Evangelisti et al. (2019a) outlined the intricate nature of nuclear movement in *Phytophthora* hyphae, where nuclei move at variable speeds within the same hyphal segment based on cytoplasmic flow, centrosome activity and organelle distribution. Thus, the ability to visualize *P. cinnamomi* nuclei with Hoechst stain provides opportunities to delve into the dynamics of nuclear movement within this pathogen.

Hardham (1987) conducted initial investigations into mitochondrial distribution in *P. cinnamomi*, and found that in zoospores the mitochondria are confined to the sub peripheral zone of the cytoplasm. Contemporary research has shifted towards the exploration of mitochondrial genome studies (Yuan et al. 2017; Makkonen et al. 2016), emphasizing the enduring significance of mitochondria. Thus, through our study, we confirm the use of MitoTracker to visualize oomycete mitochondria and, affirm the presence of these vital organelles at the periphery of encysting zoospores and their distribution throughout the hyphae, especially in actively growing regions, such as growing hyphal tips and hyphal branches.

Apart from visualization of asexual structures of *P. cinnamomi*, we have identified that the three dyes-FDA, Hoechst and MitoTracker-can be used to understand protoplast formation and composition. The primary benefit of using these fluorescent dyes includes the ability to quantify viable/non-viable protoplasts and understand their cellular makeup through image analysis and FCM. While zoospores, the most infective propagule of the pathogen (Cahill et al. 1996), are the result of sporangial programmed cell division and organelle distribution, the mechanism responsible for the partitioning of these important organelles into a protoplast has not been investigated. In our study, through the use of Hoechst stain, we found that *P. cinnamomi* protoplasts have varying numbers of nuclei. Unlike the synchronous nuclear division observed in multinucleated plant protoplasts (Motoyoshi 1971), we lack information regarding the behaviour of multinucleated *Phytophthora* protoplasts. In the absence of a genetic repository, the protoplasts do not survive. Simultaneously, multinucleated protoplasts are primarily a disadvantage during transformation as the nuclei within the same protoplast may be altered differently, resulting in a heterokaryotic population. Similarly, the distribution of mitochondria within protoplasts varies, and drawing connections between our findings and previous investigations of *P. cinnamomi*, we noted a resemblance in the cellular composition of protoplasts with that of oospores (sexual structures), and chlamydospores (dormant, asexual structures). All of these structures appear to exhibit a prominent ratio of ‘storage’ materials, such as vacuoles containing dense inclusions and lipid-like bodies, as opposed to ‘functional’ cytoplasmic elements like nuclei, mitochondria and ribosomes (Hemmes and Wong 1975).

Flow cytometry is commonly used to assess and quantify the fluorescent properties of plant protoplasts (Bargmann and Birnbaum 2009), but has been seldom used in oomycete studies. For example, FCM was able to differentiate *P. infestans* from other potential airborne particles (Day et al. 2002), while in other studies, FCM has been used to measure the nuclear DNA content of the pathogen (Catal et al. 2010; Silva et al. 2021). Through our study, we confirm that FCM is a powerful tool to analyze *P. cinnamomi* protoplasts in conjunction with the organelle-specific fluorescent stains. FCM allows us to understand a large population of protoplasts at once and sort them into specific groups, based on the fluorescent labels used (Zhou et al. 2019). We have shown novel applications of the dyes in an oomycete system and verified the specific labelling of cellular structures. Hence, these techniques along with other organelle specific dyes can be exploited to better understand the cytoplasmic rerouting that occurs within the oomycete hypha, spores, and protoplasts.

### Improved understanding of protoplast isolation and regeneration

Obtaining protoplasts in sufficient quantity and of adequate quality is a major challenge with *Phytophthora* species, mainly due to high protoplast variability and low viability. We have hereby optimized the different steps crucial in protoplast isolation and show the feasibility of using these protoplasts for transformation studies.

#### Germinated cysts of coenocytic *P. cinnamomi* generate viable protoplasts

Protoplasts are spherical, membrane bound bodies, that lack the cell wall and are osmotically sensitive (de Jimenez et al. 1979). *Phytophthora* mycelium is multi-branched and coenocytic, allowing free flow of the cytoplasmic contents. Thus, protoplasts liberated from hyphae will have variations in the cytoplasmic content (Bartnicki-Garcia 1966) (Supplementary Video 1). In previous studies with *P. parasitica* (Jahnke et al. 1987) and *P. capsici* (Yi et al. 1993), young mycelia was used for protoplast isolation (Jahnke et al. 1987). The influence of mycelial age on cell wall composition and distribution of the cytoplasmic content has been established for several species of *Phytophthora* (Duan et al. 2011). In more mature cultures, the cellular content is spread across multi-branched hyphae and as a result, there is a lower ratio of nuclear and cytoplasmic content to the mycelial volume (Wagner and Wilkinson 1993; Howlett 1989). In contrast, in germinated cysts, there is much less hyphal branching, cytoplasmic flow is limited, and nuclei and cell organelles are restricted in their distribution, thus, producing more viable protoplasts.

We have found that for *P. cinnamomi* using pea broth results in rapid vegetative growth, abundant sporangia production and consistent release of zoospores. In other studies, protoplast liberation from *Phytophthora* species has also been shown to be dependent on the type of media used for growth of the starting material (McLeod et al. 2008; Ah-Fong and Judelson 2011). In our present study with *P. cinnamomi,* the use of pea broth with β-sitosterol yielded the highest number of protoplasts. The higher number of protoplasts may be a result of increased nutrient uptake and as suggested by Calderone and Norman (1976), a consequent alteration in the structural components of the cell membrane and/or precursors for signal transduction.

#### Regeneration of protoplasts derived from germinated cysts requires an osmoticum

Low regeneration frequency of protoplasts is an ongoing challenge for those dealing with oomycetes, and remains somewhat of a challenge in protoplast-based plant research (Reed and Bargmann 2021). It has been recognized for oomycetes that the use of an osmoticum (such as mannitol or sorbitol) allows preplasmolysis of the starting material to occur, preventing damage of the cell membrane (Vessabutr and Grant 1995; McLeod et al. 2008). An osmotic stabilizer is frequently used during protoplast isolation to maintain internal and external pressure on the membrane-bound cell, to support enzymatic hydrolysis and prevent protoplast rupture (Bartnicki-Garcia 1966; Zhao et al. 2004; Yao et al. 2016). Researchers have used mannitol during the initial stages of protoplast regeneration, in *Phytophthora* species (Horta et al. 2008; Dunn et al. 2013; Gu et al. 2021). At low mannitol concentrations, the protoplasts experience low exterior solution pressure, resulting in a reduced rate of protoplast regeneration (Zhao et al. 2004; Yao et al. 2016). At mannitol concentrations higher than 0.5 M, the protoplasts either ruptured due to high exterior solution pressure or they exhibited morphological differences, such as hyphal swelling and shorter hyphal growth (Ruesink 1978). Abnormal hyphal swellings due to osmotic pressure was observed in protoplasts regenerated from *P. parasitica* (Jahnke et al. 1987), and a similar effect was noted with yeasts and filamentous fungi (Nečas and Svoboda 1985). We have found in our study that a medium with 0.5 M mannitol is optimally suited for *P. cinnamomi* protoplast regeneration.

### Transformation of *P. cinnamomi* protoplasts isolated from germinated cysts

In the present study, a high yield of protoplasts was obtained from which transformants expressing cyan fluorescence were derived. We observed through subculturing on media with a selectable marker (G418) that some putative transformants lost the ability to be antibiotic resistant. Our study demonstrates that *P. cinnamomi* like other members of the genus can rapidly adapt to their growing conditions, as observed for example, in a fungicidal study (phosphite application) (Hunter et al. 2022) and a gene editing study (Wang et al. 2019) that targeted the oxysterol binding protein. When left in their niches, *Phytophthora* species conserve their genotype and phenotype, however, changes in their environment and/or host forces them to alter their genotype and/or phenotype.

Heterokaryosis is the hallmark of *Phytophthora* species and hence, understanding the genetics of these organisms have been difficult (Catal et al. 2010; Kasuga et al. 2016; Knaus et al. 2020). Reports of polyploidy and genetic flexibility has further complicated the process of understanding the genetic makeup of *Phytophthora* species. *P. infestans* has a fluid and adaptable genome with capability to shift the ploidy number, adjust gene content and alter transcription patterns, making it a highly successful plant pathogen (Matson et al. 2022). Previously considered to be a diploid organism (Brasier and Sansome 1975), a study by Engelbrecht et al. (2021) confirmed that *P. cinnamomi* has a triploid genome with varying levels of aneuploidy and has a heterozygosity level of 1.36%. Hence, the genetic variability within *P. cinnamomi* further adds to the conundrum of difficulties associated in working with this species.

Understanding the factors influencing the generation of stable transformants remains a challenge following transformation, as it involves a complex interplay of various factors such as the fate of foreign DNA, selection of media, and the host genome. In both *P. infestans* (Judelson and Whittaker 1995) and *P. cinnamomi* (Horta et al. 2008), stable integration of marker genes has been observed; however, long-term monitoring has revealed a gradual loss of selection activity. Factors such as abortive transformation and phenotypic plasticity have been identified as contributors leading to unstable transformation outcomes. Studies on other organisms have shown that the fate of incoming DNA is crucial during transformation, with potential factors including autonomous replication of plasmids, binding to important host gene sites, and severe rearrangements of the foreign DNA over time, that may contribute to success or failure of stable transformation (Mach 2003; San Millan and MacLean 2017). Therefore, it is imperative to delve deeper into these complexities to enhance our understanding and improve the efficiency of stable transformation processes.

## Conclusion

In the present study, we established a simple, inexpensive, and efficient system to transform *P. cinnamomi* using germinated cysts, developed from zoospores. Considering that protoplast-based transformation serves as the predominant approach for gene editing in oomycetes, we have developed a robust protocol for isolation of viable protoplasts of *P. cinnamomi*. To our knowledge, this is the first work to outline the use of microscopy and FCM along with cell-specific dyes to visualize organelle distribution within *P. cinnamomi* protoplasts. The methodologies we have described can serve as a foundation for developing protocols for transformation in other, less studied *Phytophthora* species. It is crucial to continue our studies on gene functionalisation in *P. cinnamomi*, as one of the most challenging plant pathogens. There is also an urgency to understand the pathogenicity of *P. cinnamomi* through transformation approaches in order to develop new targets for disease management.

### Supplementary Information

Below is the link to the electronic supplementary material.

Supplementary file1 Supplementary Fig. 1 Validation of plasmid, pCFPN, through restriction enzyme digest and PCR (a) Plasmid map of pCFPN containing cyan fluorescent protein gene (*CFP*) along with neomycin phosphotransferase gene (*nptII*). The plasmid contains transcriptional regulators from *Bremia lactucae*, where *ham34 *is flanked to *CFP*, and *hsp70 *is flanked to the geneticin (G418)-resistant gene, *nptII*. The plasmid also contains ampicillin resistant gene (*amp*). (b) Restriction enzyme digest of the extracted plasmid with *NheI *and *BsaI*. L, Hyperladder™ 1 kb, 1, undigested plasmid, pCFPN, 2, digested plasmid with *NheI *and *BsaI *(4844 bp, 2128 bp). (c) PCR assay of *nptII *and *CFP *genes. L, Hyperladder™ 100 bp, 1, plasmid amplified with G418 primers (493 bp), 2, plasmid amplified with CFP primers (312 bp). (TIF 43.4 MB)

Supplementary file2 Supplementary Fig. 2 Enzymatic digestion of *P. cinnamomi *mycelia before/after incubation for 45 minutes in the dark with shaking at 55 rpm (a – c) shows mycelia harvested after incubation in pea broth with β-sitosterol, for varying durations. (d) Germinated cysts of *P. cinnamomi *incubated in pea broth with β-sitosterol for 18 hours. The starting material was placed in 5 mg/mL of cellulase and lysing enzyme to initiate enzymatic digestion. (e-h) shows the result after enzymatic digestion of the starting material. In (e) and (h), the starting material has digested completely, leaving the initial agar plugs in (e). In (f) and (g) the majority of the mycelia remains undigested due to the thick mat formed by the starting material. (Scale = 1 cm) (JPG 174 KB)

Supplementary file3 Supplementary Fig. 3 Summary of the percentage of *P. cinnamomi *protoplasts that have regenerated in different mannitol concentrations. Protoplasts were isolated from mature hyphae (18 or 40 hours) and germinated cysts (18 hours) grown in different medium [10% cV8, pea and rich pea broth, without (-) or with (+) β-sitosterol]. The protoplasts were regenerated in pea broth supplemented with varying mannitol concentrations. There was no regeneration in 0 M mannitol. (a) 0.1 M mannitol (b) 0.3 M mannitol (c) 0.5 M mannitol (d) 0.7 M mannitol (e) 0.9 M mannitol. The highest protoplast regeneration occurred in media supplemented with 0.5 M mannitol. (JPG 117 KB)

Supplementary file4 Supplementary Fig. 4 Microscopic images of regenerated protoplasts of *P. cinnamomi* in pea broth containing mannitol concentrations ranging from 0 to 0.9 M mannitol. (a) No mannitol – no regeneration of protoplasts (b) 0.1 M mannitol – low number of protoplast regeneration, with irregular hyphae formation (c) 0.3 M mannitol – low number of protoplast regeneration with short hyphal length. (d) 0.5 M mannitol – highest number of protoplast regeneration. Healthy hyphae with cytoplasmic content observed under the microscope. (e) 0.7 M mannitol and (f) 0.9 M mannitol – moderate regeneration of protoplast, hyphal swelling observed. (Scale = 20 μm) (PNG 214 KB)

Supplementary file5 Supplementary Fig. 5 Determination of the minimum inhibitory concentration (MIC) of geneticin (also known as G418) on growth of *P. cinnamomi *(a) Growth of mature *P. cinnamomi *(grown from plugs) in 10% cV8 agar supplemented with varying concentrations of G418. The MIC in this media is 30 μg/mL. (c) Growth of mature *P. cinnamomi *(grown from plugs) in pea agar supplemented with varying concentrations of G418. The MIC in this media is 40 μg/mL. (e) Regeneration of protoplasts in pea agar with varying concentrations of G418. The MIC in this media is 20 μg/mL. (b) and (d) are microscope images of *P. cinnamomi *at the respective G418 concentrations. All Petri plates are of 90 mm diameter. (Scale for microscope images = 50 μm) (TIF 21775 KB)

Supplementary file6 Supplementary Fig. 6 Summary of regenerants obtained after transformation of *P. cinnamomi *with plasmid, pCFPN (a) Summary of the distribution of *P. cinnamomi *putative transformants after being subcultured on 20 μg/mL G418, twice. (b) Pictorial representation of the ratings provided to each of the putative transformants when subcultured on pea broth with 20 μg/mL G418. (c) Day 5 images of growth of the three positive transformants (Pc-CFP-1, Pc-CFP-2 and Pc-CFP-4) from each replicate, on pea broth without or with 20 μg/mL G418 after subsequent subcultures. Wildtype *P. cinnamomi *is labelled as Pc-WT. (TIF 6624 KB)

Supplementary file7 Supplementary Table 1 Primer pairs and PCR conditions used for amplification of the selective marker, *nptII*, the visual reporter, *CFP*, and the internal control for *P. cinnamomi*, *actin *(DOCX 14.5 KB)
Supplementary Video 1 Formation of protoplast from the coenocytic, *P. cinnamomi *hyphae during enzyme digestion. The motion of free-flowing cytoplasm in the hyphae is visible, though the driving forces behind the movement is unknown. Protoplast formation is observed in boxes a and b, as the cell wall is digested by cellulase and lysing enzymes. The video is a time-lapse comprising 61 frames captured over a duration of 599 seconds in real-time. (Scale = 20 μm) (DOCX 15 KB)

Supplementary file8 (AVI 29112 KB)
