# Supplementary figures and images for: Viable protoplast isolation, organelle visualization and transformation of the globally distributed plant pathogen Phytophthora cinnamomi
Source: Protoplasma. 2024 May 4;261(5):1073–92. doi: 10.1007/s00709-024-01953-y (PMC11358197; doi:10.1007/s00709-024-01953-y)

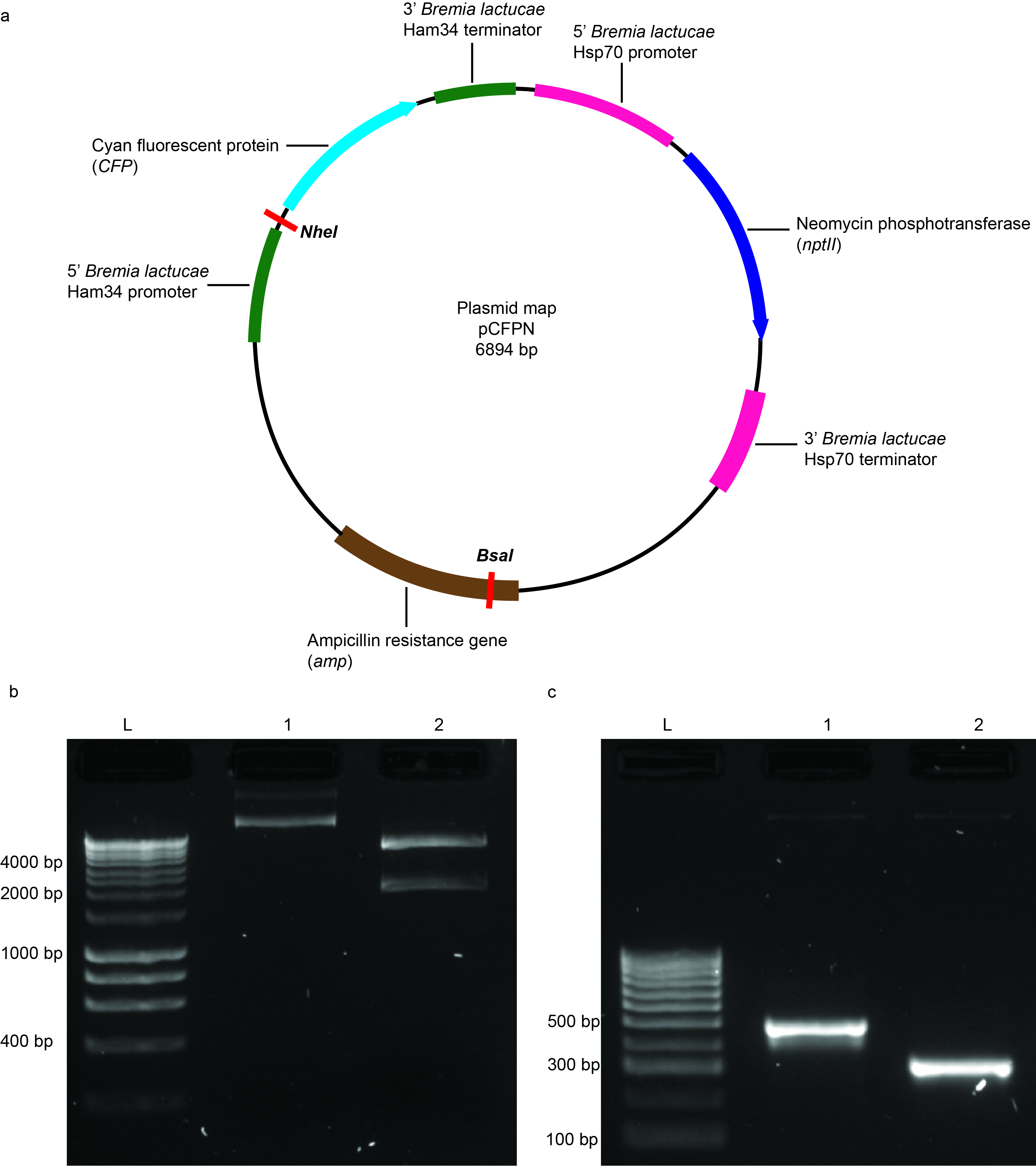

Supplement: Supplementary file 1 — Supplementary file1 Supplementary Fig. 1 Validation of plasmid, pCFPN, through restriction enzyme digest and PCR (a) Plasmid map of pCFPN containing cyan fluorescent protein gene (CFP) along with neomycin phosphotransferase gene (nptII). The plasmid contains transcriptional regulators from Bremia lactucae, where ham34 is flanked to CFP, and hsp70 is flanked to the geneticin (G418)-resistant gene, nptII. The plasmid also contains ampicillin resistant gene (amp). (b) Restriction enzyme digest of the extracted plasmid with NheI and BsaI. L, Hyperladder™ 1 kb, 1, undigested plasmid, pCFPN, 2, digested plasmid with NheI and BsaI (4844 bp, 2128 bp). (c) PCR assay of nptII and CFP genes. L, Hyperladder™ 100 bp, 1, plasmid amplified with G418 primers (493 bp), 2, plasmid amplified with CFP primers (312 bp). (TIF 43.4 MB) [file 709_2024_1953_MOESM1_ESM.tif]

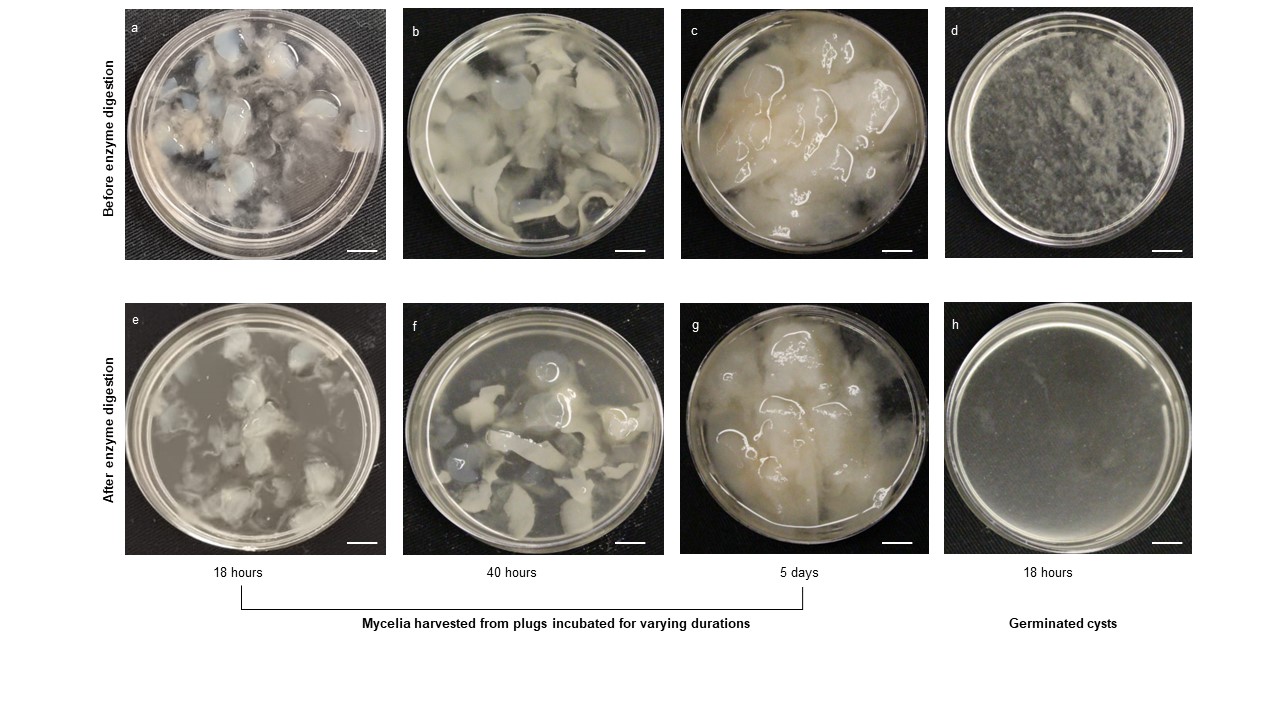

Supplement: Supplementary file 2 — Supplementary file2 Supplementary Fig. 2 Enzymatic digestion of P. cinnamomi mycelia before/after incubation for 45 minutes in the dark with shaking at 55 rpm (a – c) shows mycelia harvested after incubation in pea broth with β-sitosterol, for varying durations. (d) Germinated cysts of P. cinnamomi incubated in pea broth with β-sitosterol for 18 hours. The starting material was placed in 5 mg/mL of cellulase and lysing enzyme to initiate enzymatic digestion. (e-h) shows the result after enzymatic digestion of the starting material. In (e) and (h), the starting material has digested completely, leaving the initial agar plugs in (e). In (f) and (g) the majority of the mycelia remains undigested due to the thick mat formed by the starting material. (Scale = 1 cm) (JPG 174 KB) [file 709_2024_1953_MOESM2_ESM.jpg]

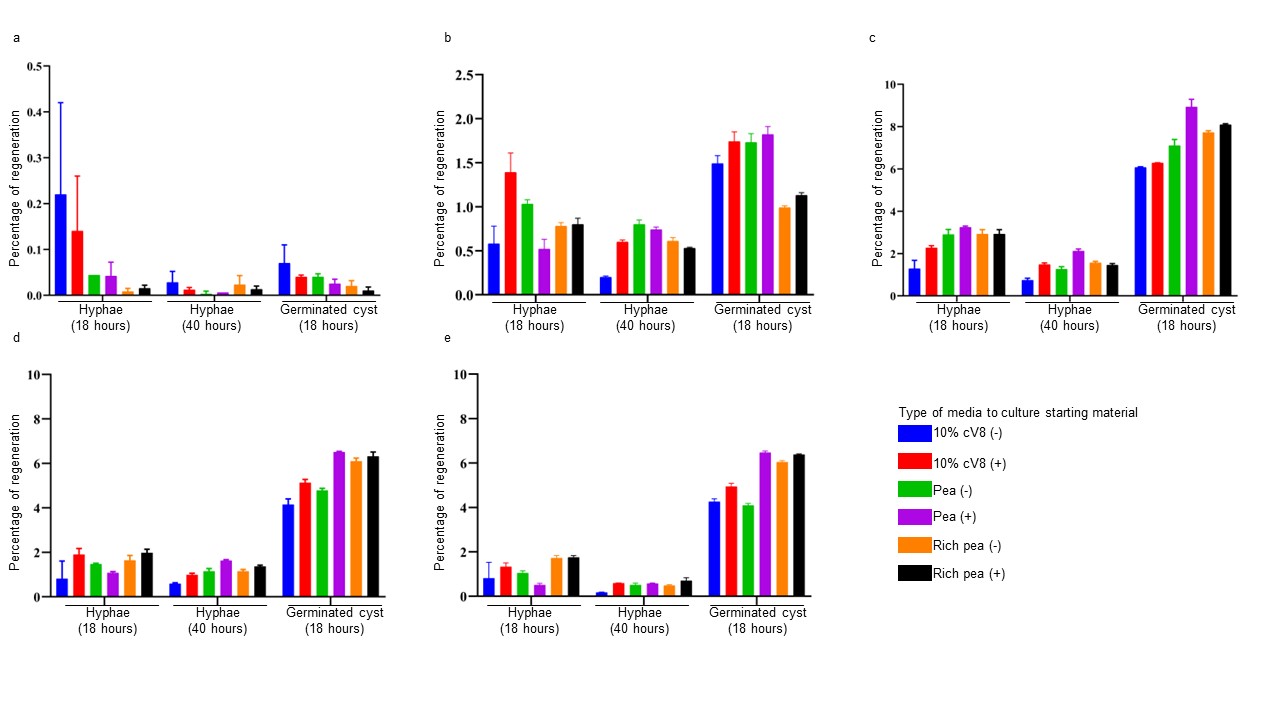

Supplement: Supplementary file 3 — Supplementary file3 Supplementary Fig. 3 Summary of the percentage of P. cinnamomi protoplasts that have regenerated in different mannitol concentrations. Protoplasts were isolated from mature hyphae (18 or 40 hours) and germinated cysts (18 hours) grown in different medium [10% cV8, pea and rich pea broth, without (-) or with (+) β-sitosterol]. The protoplasts were regenerated in pea broth supplemented with varying mannitol concentrations. There was no regeneration in 0 M mannitol. (a) 0.1 M mannitol (b) 0.3 M mannitol (c) 0.5 M mannitol (d) 0.7 M mannitol (e) 0.9 M mannitol. The highest protoplast regeneration occurred in media supplemented with 0.5 M mannitol. (JPG 117 KB) [file 709_2024_1953_MOESM3_ESM.jpg]

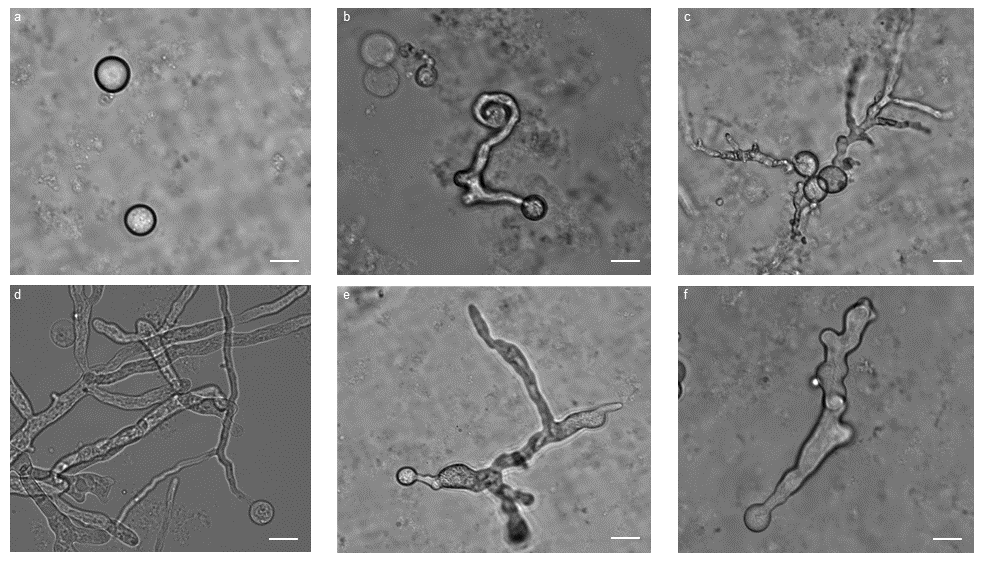

Supplement: Supplementary file 4 — Supplementary file4 Supplementary Fig. 4 Microscopic images of regenerated protoplasts of P. cinnamomi in pea broth containing mannitol concentrations ranging from 0 to 0.9 M mannitol. (a) No mannitol – no regeneration of protoplasts (b) 0.1 M mannitol – low number of protoplast regeneration, with irregular hyphae formation (c) 0.3 M mannitol – low number of protoplast regeneration with short hyphal length. (d) 0.5 M mannitol – highest number of protoplast regeneration. Healthy hyphae with cytoplasmic content observed under the microscope. (e) 0.7 M mannitol and (f) 0.9 M mannitol – moderate regeneration of protoplast, hyphal swelling observed. (Scale = 20 μm) (PNG 214 KB) [file 709_2024_1953_MOESM4_ESM.png]

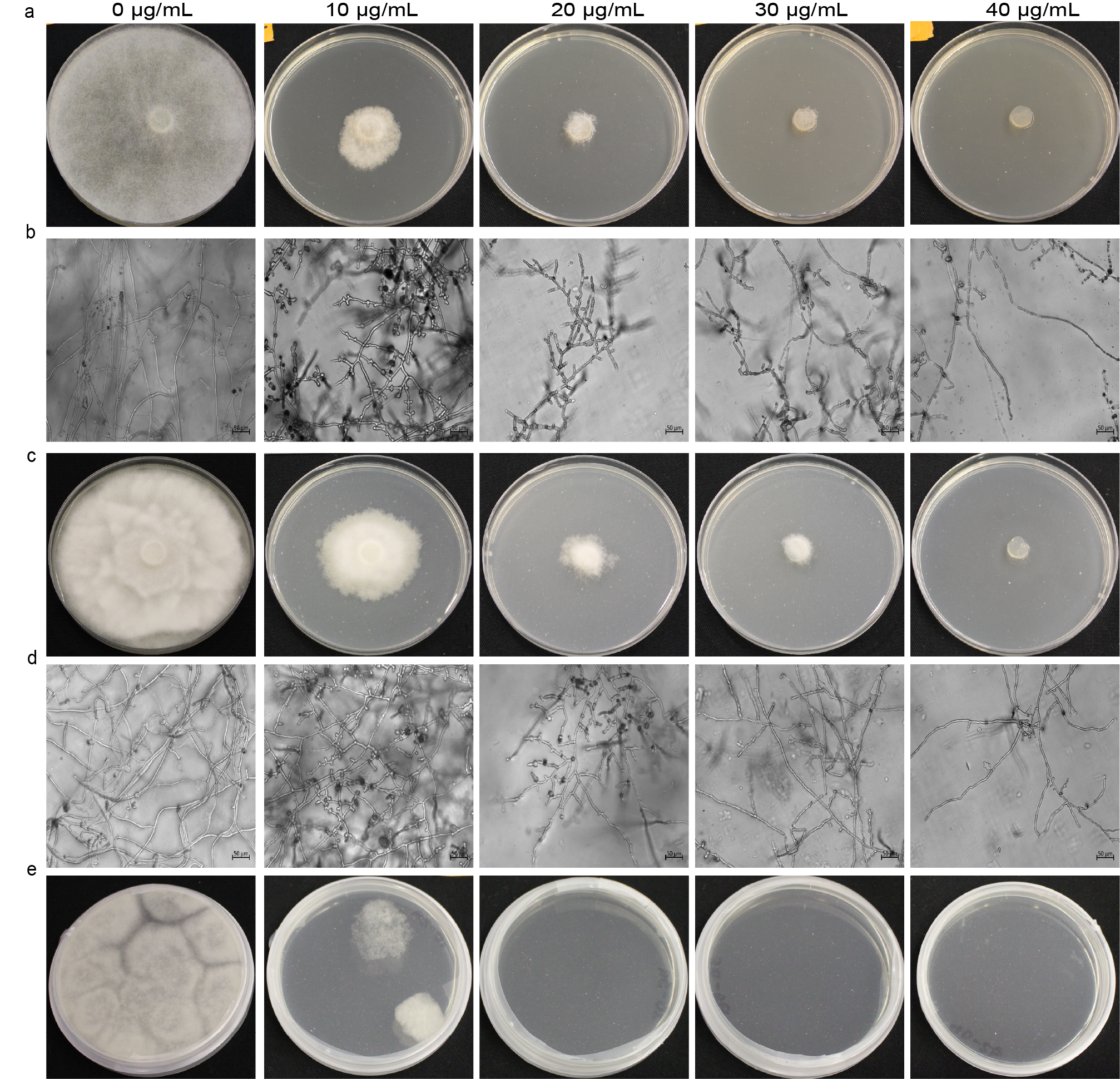

Supplement: Supplementary file 5 — Supplementary file5 Supplementary Fig. 5 Determination of the minimum inhibitory concentration (MIC) of geneticin (also known as G418) on growth of P. cinnamomi (a) Growth of mature P. cinnamomi (grown from plugs) in 10% cV8 agar supplemented with varying concentrations of G418. The MIC in this media is 30 μg/mL. (c) Growth of mature P. cinnamomi (grown from plugs) in pea agar supplemented with varying concentrations of G418. The MIC in this media is 40 μg/mL. (e) Regeneration of protoplasts in pea agar with varying concentrations of G418. The MIC in this media is 20 μg/mL. (b) and (d) are microscope images of P. cinnamomi at the respective G418 concentrations. All Petri plates are of 90 mm diameter. (Scale for microscope images = 50 μm) (TIF 21775 KB) [file 709_2024_1953_MOESM5_ESM.tif]

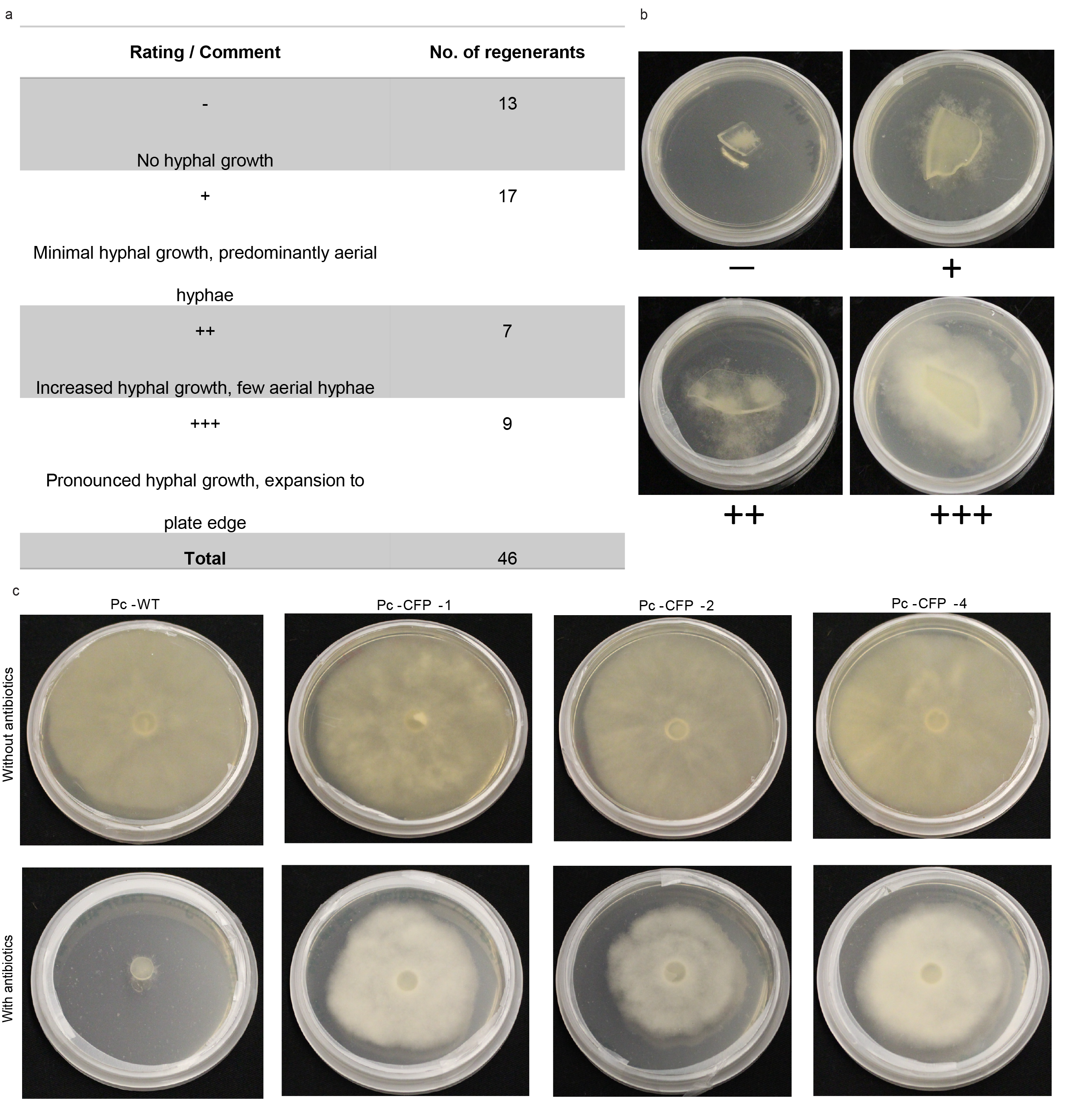

Supplement: Supplementary file 6 — Supplementary file6 Supplementary Fig. 6 Summary of regenerants obtained after transformation of P. cinnamomi with plasmid, pCFPN (a) Summary of the distribution of P. cinnamomi putative transformants after being subcultured on 20 μg/mL G418, twice. (b) Pictorial representation of the ratings provided to each of the putative transformants when subcultured on pea broth with 20 μg/mL G418. (c) Day 5 images of growth of the three positive transformants (Pc-CFP-1, Pc-CFP-2 and Pc-CFP-4) from each replicate, on pea broth without or with 20 μg/mL G418 after subsequent subcultures. Wildtype P. cinnamomi is labelled as Pc-WT. (TIF 6624 KB) [file 709_2024_1953_MOESM6_ESM.tif]
