# Supplementary material for: Viable protoplast isolation, organelle visualization and transformation of the globally distributed plant pathogen Phytophthora cinnamomi
Source: Protoplasma. 2024 May 4;261(5):1073–92. doi: 10.1007/s00709-024-01953-y (PMC11358197; doi:10.1007/s00709-024-01953-y)
Supplement: Supplementary file 7 — Supplementary file7 Supplementary Table 1 Primer pairs and PCR conditions used for amplification of the selective marker, nptII, the visual reporter, CFP, and the internal control for P. cinnamomi, actin (DOCX 14.5 KB) Supplementary Video 1 Formation of protoplast from the coenocytic, P. cinnamomi hyphae during enzyme digestion. The motion of free-flowing cytoplasm in the hyphae is visible, though the driving forces behind the movement is unknown. Protoplast formation is observed in boxes a and b, as the cell wall is digested by cellulase and lysing enzymes. The video is a time-lapse comprising 61 frames captured over a duration of 599 seconds in real-time. (Scale = 20 μm) (DOCX 15 KB) [file 709_2024_1953_MOESM7_ESM.docx]

Supplementary Table 1 Primer pairs and PCR conditions used for amplification of selective marker, *nptII*, visual reporter, *CFP* and internal control for *P. cinnamomi*, *actin*.

| **Primer** | Sequence | Expected band size |
| --- | --- | --- |
| FPN_For | 5’ TGACCCTGAAGTTCATCTGCACC 3’ | 312 bp |
| FPN_Rev | 5’ GGCACAAGCTGGAGTACAACTACA 3’ |  |
| G418_For | 5’ TCTTTTTGTCAAGACCGACCTGT 3’ | 493 bp |
| G418_Rev | 5’ TTTCTGGATTCATCGACTGTGGC 3’ |  |
| Actin_For | 5’ GGCGAGCGTATGACGAAGGA 3’ | 124 bp |
| Actin_Rev | 5’ TCGACAGCGACGACAGGATG 3’ |  |
